# Functional changes in the oral microbiome after use of fluoride and arginine containing dentifrices: a metagenomic and metatranscriptomic study

**DOI:** 10.1186/s40168-022-01338-4

**Published:** 2022-09-28

**Authors:** Miguel Carda-Diéguez, Rebecca Moazzez, Alex Mira

**Affiliations:** 1 Genomics and Health Department, FISABIO Institute, Valencia, Spain; 2 Faculty of Dentistry, Oral & Craniofacial Sciences, King’s College London, London, UK; 3 Network of Epidemiology and Public Health, CIBERESP, Madrid, Spain

**Keywords:** Oral microbiota, Caries, Arginine, Fluoride, Arginolytic route, Metatranscriptome, Metagenome, 16S rRNA

## Abstract

**Background:**

Tooth decay is one of the most prevalent diseases worldwide, and efficient tooth brushing with a fluoride-containing dentifrice is considered fundamental to caries prevention. Fluoride-containing dentifrices have been extensively studied in relation to enamel resistance to demineralization. Arginine (Arg) has also been proposed as a promising prebiotic to promote pH buffering through ammonia production. Here, we present the first metagenomic (DNA sequencing of the whole microbial community) and metatranscriptomic (RNAseq of the same community) analyses of human dental plaque to evaluate the effect of brushing with fluoride (Fl) and a Fl+Arg containing dentifrices on oral microbial composition and activity. Fifty-three patients were enrolled in a longitudinal clinical intervention study with two arms, including 26 caries-active and 27 caries-free adults. After a minimum 1-week washout period, dental plaque samples were collected at this post-washout baseline, 3 months after the use of a 1450-ppm fluoride dentifrice, and after 6 months of using a 1450-ppm fluoride with 1.5% arginine dentifrice.

**Results:**

There was a shift in both the composition and activity of the plaque microbiome after 3 months of brushing with the fluoride-containing toothpaste compared to the samples collected at the 1-week post-washout period, both for caries-active and caries-free sites. Although several caries-associated bacteria were reduced, there was also an increase in several health- and periodontitis-associated bacteria. Over 400 genes changed proportion in the metagenome, and between 180 and 300 genes changed their expression level depending on whether caries-free or caries-active sites were analyzed. The metagenome and metatranscriptome also changed after the subjects brushed with the Fl+Arg dentifrice. There was a further decrease of both caries- and periodontitis-associated organisms. In both caries-free and caries-active sites, a decrease of genes from the arginine biosynthesis pathway was also observed, in addition to an increase in the expression of genes associated with the arginine deiminase pathway, which catabolizes arginine into ammonia, thereby buffering acidic pH. Bacterial richness and diversity were not affected by either of the two treatments in the two arms of the study.

**Conclusions:**

Our data demonstrate that long-term use of both assayed dentifrices changes the bacterial composition and functional profiles of human dental plaque towards a healthier microbial community, both in caries-free and caries-active sites. This observation was especially apparent for the Fl+Arg dentifrice. Thus, we conclude that the preventive benefits of tooth brushing go beyond the physical removal of dental plaque and that the active ingredients formulated within dentifrices have a positive effect not only on enamel chemistry but also on the metabolism of oral microbial populations.

Video Abstract

**Supplementary Information:**

The online version contains supplementary material available at 10.1186/s40168-022-01338-4.

## Background

Caries is a multi-factorial disease caused by microbial-derived acidic pH, which demineralizes the enamel [1]. It is considered one of the most widespread diseases worldwide, and efficient tooth brushing with a fluoride dentifrice is fundamental to caries prevention [2–5]. Several microbiological studies have proven a direct role for oral microorganisms in enamel demineralization, through the fermentation of dietary sugars [6–10]. Fluoride-containing dentifrices have been extensively studied in relation to enamel resistance to demineralization with solid epidemiological evidence, but its effect on the oral microbiota is less understood [11–14]. For example, it has been shown that fluoride inhibits microbial glucolytic activity, but the current evidence is controversial [12, 15]. In addition to fluoride, arginine has also been shown to be an effective anti-caries agent [16]. Dentifrice formulations containing fluoride and arginine have similarly been shown to provide further protection against caries initiation and progression [17–22]. The reason is that many beneficial oral bacteria are able to transform arginine into ammonia through the arginine deiminase pathway (ADS), which is a base that effectively buffers acidic pH [23]. Thus, making arginine available to the oral microbial community is expected to stimulate arginolytic organisms, thereby increasing the biofilm’s buffering capacity and reducing its acidogenicity [24]. However, there is currently a lack of in vivo studies evaluating the effect of arginine on periodontal pathogens.

Although there is solid clinical evidence of the beneficial effects of fluoride and arginine dentifrices, the availability of functional studies that evaluate the bacterial composition and activity in vivo is scarce. Regarding this, the new “omics” techniques now allow researchers to functionally characterize a microbial community [25] based on the full genetic potential (a metagenomics approach, by which the whole DNA is directly sequenced) or based on the gene expression profile (a metatranscriptomic approach, by which the whole RNA is retrotranscribed to cDNA and then sequenced). In relation to caries, the potential of these holistic OMICS approaches has been shown in the last years by characterizing the metabolome of saliva after a sugar rinse [12], by describing the metagenome and metatranscriptome of caries lesions [8, 10], by comparing the metaproteome of dental plaque in caries-free and caries-active individuals [26], or by identifying the genes expressed by microbes during dental plaque development [27].

**Fig. 1:**
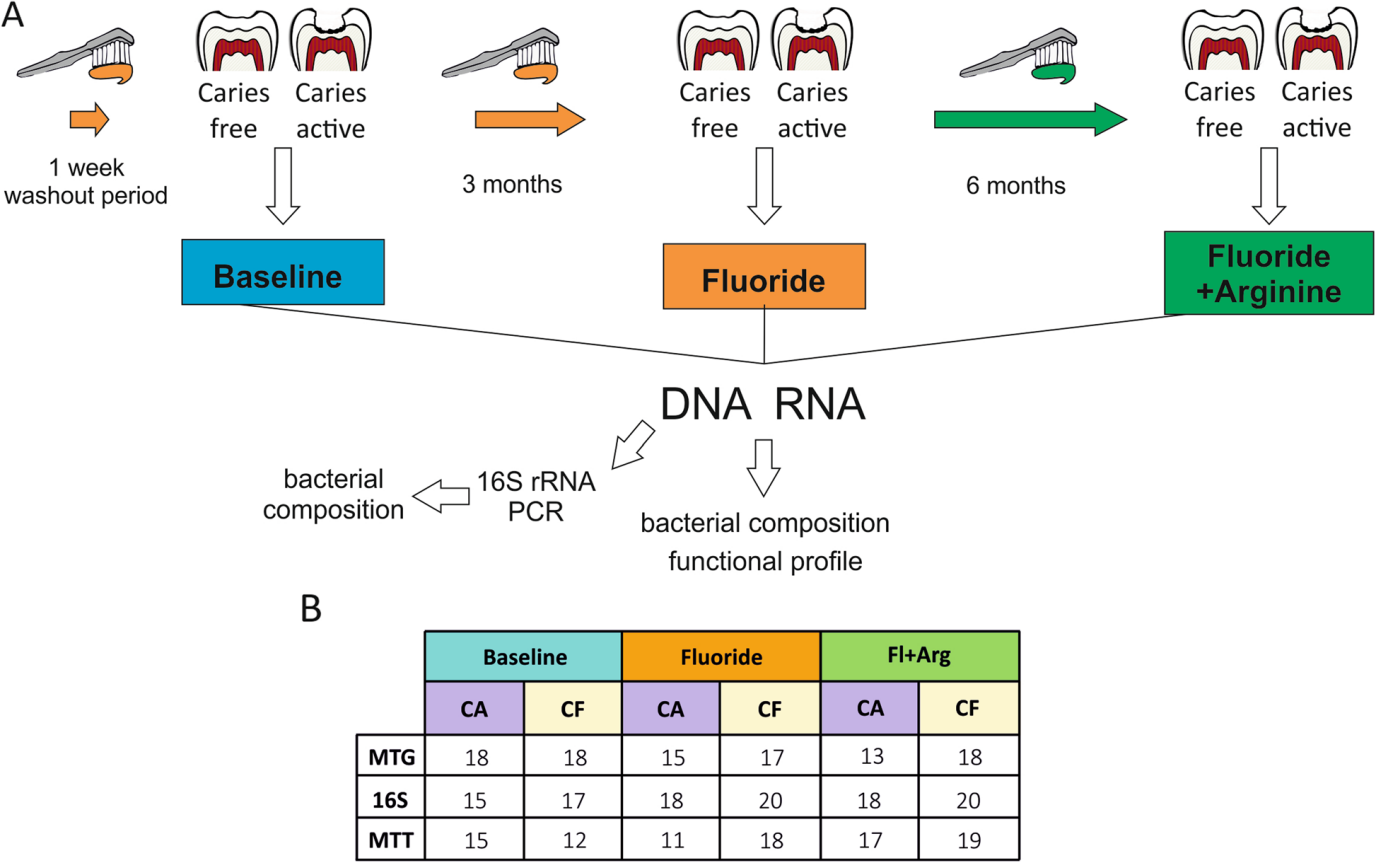
Structure of the longitudinal clinical intervention study. **A** After a 1-week washout period, individuals brushed their teeth twice per day for a period of 3 months with a 1450-ppm fluoride dentifrice and six additional months with a 1.5% arginine + 1450 ppm fluoride dentifrice. Dental plaque samples were collected longitudinally at post-washout (marked as “Baseline” in the figure) and after the two treatment periods (arrows). DNA and RNA were extracted from the plaque samples and separately sequenced on an Illumina platform. The corresponding metagenome (MTG) and metatranscriptome (MTT) were analyzed to determine the bacterial composition, gene content, and gene expression of the overall dental plaque community. In addition, the 16S rRNA gene was amplified in the DNA samples and sequenced separately through Illumina Miseq sequencing. **B** Number of dental plaque samples analyzed in the current manuscript for the two arms of the study, i.e., caries-active (CA) and caries-free (CF) individuals at the three time points. Numbers in the table represent the final number of samples included in the analysis after excluding patients that abandoned the study and samples with low number of reads. MTG, metagenomic (i.e., DNA) dataset; MTT, metatranscriptomic (i.e., RNA) dataset; 16S (i.e., amplified DNA) dataset

In the current work, subjects were given a new toothbrush and instructed to brush 2×/day with a fluoride-containing dentifrice for a week to homogenize the differences in oral microbiota caused by the differences in oral hygiene habits and dentifrice use prior to the study. Then, they were asked to brush with the same fluoride dentifrice for 3 months to further harmonize the microbiota before introduction to the Fl+Arg dentifrice. The Fl+Arg dentifrice was used for the remaining 6 months of the study to investigate the additional effects of arginine over the microbiota (Fig. 1). In this paper, we describe for the first time the metagenome (16S rRNA and whole-DNA shotgun sequencing) and metatranscriptome of dental plaque microbiota before and after long-term use of fluoride and also a Fl+Arg-containing dentifrice. Using this clinical design, it was possible to explore shifts in microbial composition, as well as to study changes in genetic content and gene expression patterns, with the purpose of monitoring bacterial functional activity and understanding how they mediate oral health.

## Results

The effect of fluoride and Fl+Arg dentifrices over the taxonomic composition, genetic potential, and functional activity of the oral microbiota was studied in 26 caries-active (CA) and 27 caries-free (CF) patients (Fig. 1A). Fifteen patients dropped off the study before all visits were completed. Eighteen CA and 20 CF subjects completed the study. After a minimum 1-week washout period, dental plaque was sampled (post-washout *baseline*) and patients were asked to continue toothbrushing with the dentifrice with fluoride for 3 months after which the same teeth were sampled again (*fluoride*). Participants were then asked to brush with a Fl+Arg dentifrice for 6 months, after which plaque collection was repeated at the same sites.

In order to establish a threshold to filter out low-coverage samples, we performed rarefaction analyses in the 16S rRNA sequencing (16S), the metagenome (MTG), and the metatranscriptome (MTT) datasets, by plotting the sequencing coverage against the estimated number of bacterial species, as well as to the estimated number of gene functions in the KEGG database. The curves showed that taxonomical and functional genetic richness stabilized after 10k, 100k, and 50k reads for the 16S, MTG, and MTT datasets, respectively (Additional Fig. 1). Considering that reducing the number of samples and/or including samples with a limited coverage could have an effect on the significance of the differences detected (especially those taxa/genes with low abundance), samples below those thresholds were eliminated from the MTG and MTT datasets for further analyses and paired tests could not be performed. Moreover, the high number of species detected in the MTG datasets was mostly caused by low abundant species as can be seen in rarefaction curves when only species at > 0.1% abundance were represented (Additional Fig. 1F). The final number of samples analyzed per group at each time point is shown in Fig. 1B. In these analyzed samples, the MTG datasets had an average of 4.7 × 10^6^ initial reads per sample from which 20 ± 14% were human, and 57 ± 8.7% of the remaining, bacterial reads were annotated. In the MTT datasets, an average of 5.2 × 10^6^ reads per sample were initially sequenced. Of those, 9.2 ± 11.7% belonged to the host, and 78.41 ± 20.1% of the remaining reads were ribosomal. Finally, 32.7 ± 13.2% (0.2 × 10^6^) of the remaining reads were annotated.

The oral microbiota was studied using a metagenomic and a metatranscriptomic approach, which allowed us to study not only the abundance of the different members of the microbial community but also their genetic potential and activity. For clarity, we will refer to “represented microbiota/gene abundance” when using MTG data and “active microbiota/gene expression” when reporting results from MTT data. In addition, the bacterial taxonomic composition was also confirmed by standard high-throughput Illumina sequencing of the 16S rRNA gene.

**Fig. 2:**
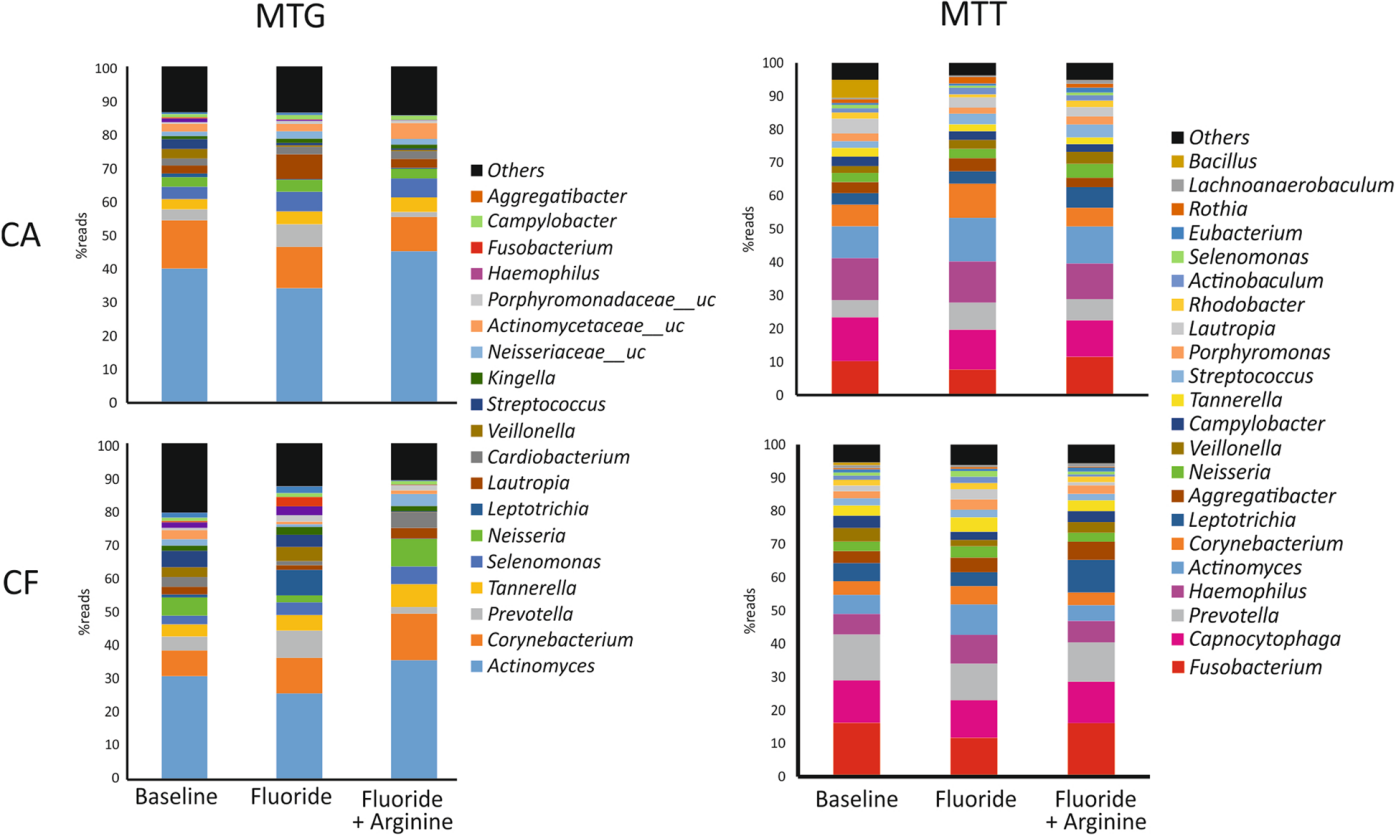
Genus composition of microbial communities associated with caries-active (CA) and caries-free (CF) individuals. Top 20 most abundant genera at baseline (post-washout period), after 3 months of treatment with fluoride dentifrice (Fluoride), and after 6 further months of treatment with fluoride + arginine (Fl+Arg) dentifrice are represented based on data from the metagenome (MTG) or metatranscriptome (MTT). Bacteria that could not be unequivocally assigned to a given genus are indicated with the name of the corresponding family followed by “uc” (unclassified)

According to MTG data, *Actinomyces* was the most abundant genus in supragingival dental plaque microbiota in both CA (39.6 ± 5.5% of the overall composition) and CF (30.3 ± 5%) patients (Fig. 2). *Corynebacterium*, *Prevotella*, *Tannerella*, *Selenomonas*, *Neisseria*, *Leptotrichia*, *Lautropia*, *Cardiobacterium*, and *Veillonella* completed the top 10 most abundant genera.

However, the annotation of MTT data suggested that the abundance of *Actinomyces* did not correlate with its activity (Fig. 2, Additional Fig. 2 and Additional Table 1). Instead, the most active genera appeared to include *Fusobacterium*, *Capnocytophaga*, *Prevotella*, *Haemophilus*, *Actinomyces*, and *Corynebacterium*, which were all present in the MTT dataset with similar percentages (5–10%) and combined accounted for 50–60% of the active microbiota. The proportion of these genera in the whole (DNA-based) and the active (RNA-based) microbiota differed substantially, suggesting that bacterial activity at a given moment may not correlate with its overall presence in the community and that its activity may change considerably through time. In fact, there was no significant correlation in bacterial composition between the MTG and the MTT datasets for the top 15 most abundant genera (*R*^2^ < 0.01 in all cases). These results are in agreement with previous reports which showed that microbial communities may significantly vary when comparing MTG and MTT data [27–29]. It must also be taken into consideration that some samples with low coverage appeared as outliers in the MTT analyses, and therefore, more consistent results could be achieved with higher sequencing coverage in that dataset.

The bacterial composition was also assessed by sequencing the 16S rRNA gene (Additional Fig. 3A). The differences between the 16S and MTG datasets may derive from the amplification step and the database used (Additional Fig. 3B). The amplification bias can favor certain microorganisms depending on the similarity of their 16S rRNA gene to the universal primers used and can also under-amplify the organisms at lower proportions [30, 31]. A dramatic case is given by *Actinomyces*, a high G+C content organism which in the MTG dataset can account for over 40% of the total (Fig. 2 and Additional Fig. 2), whereas in the 16S dataset, the levels are below 10% (Additional Fig. 3). Consequently, since 16S data is introducing a putative bias into the proportions, we considered MTG datasets more reliable and used MTG data for subsequent taxonomic description.

The 16S and MTT datasets show no change in any of the diversity indexes as a function of dentifrice treatment (Additional Fig. 4). The MTG data show a decrease in the richness indexes (ACE and Chao, which estimate the number of bacterial species) after the use of both dentifrices. This decrease was significant after the Fl+Arg treatment (*p* = 0.0016 for CF and 0.00031 for CA patients, Wilcoxon test), in agreement with previous clinical studies where an arginine-containing toothpaste was used [32]. However, a caries-free microbiota has been usually reported to be more diverse than caries-active communities. We hypothesize that after 6 months of treatment, bacteria more adapted to the arginine-rich niche are taking advantage of other microbes, reducing their abundance and consequently diversity. Diversity in gene functions did not differ between baseline and post-dentifrice use.

### Changes in plaque microbiota: baseline compared to 3 months of fluoride toothbrushing

**Fig. 3:**
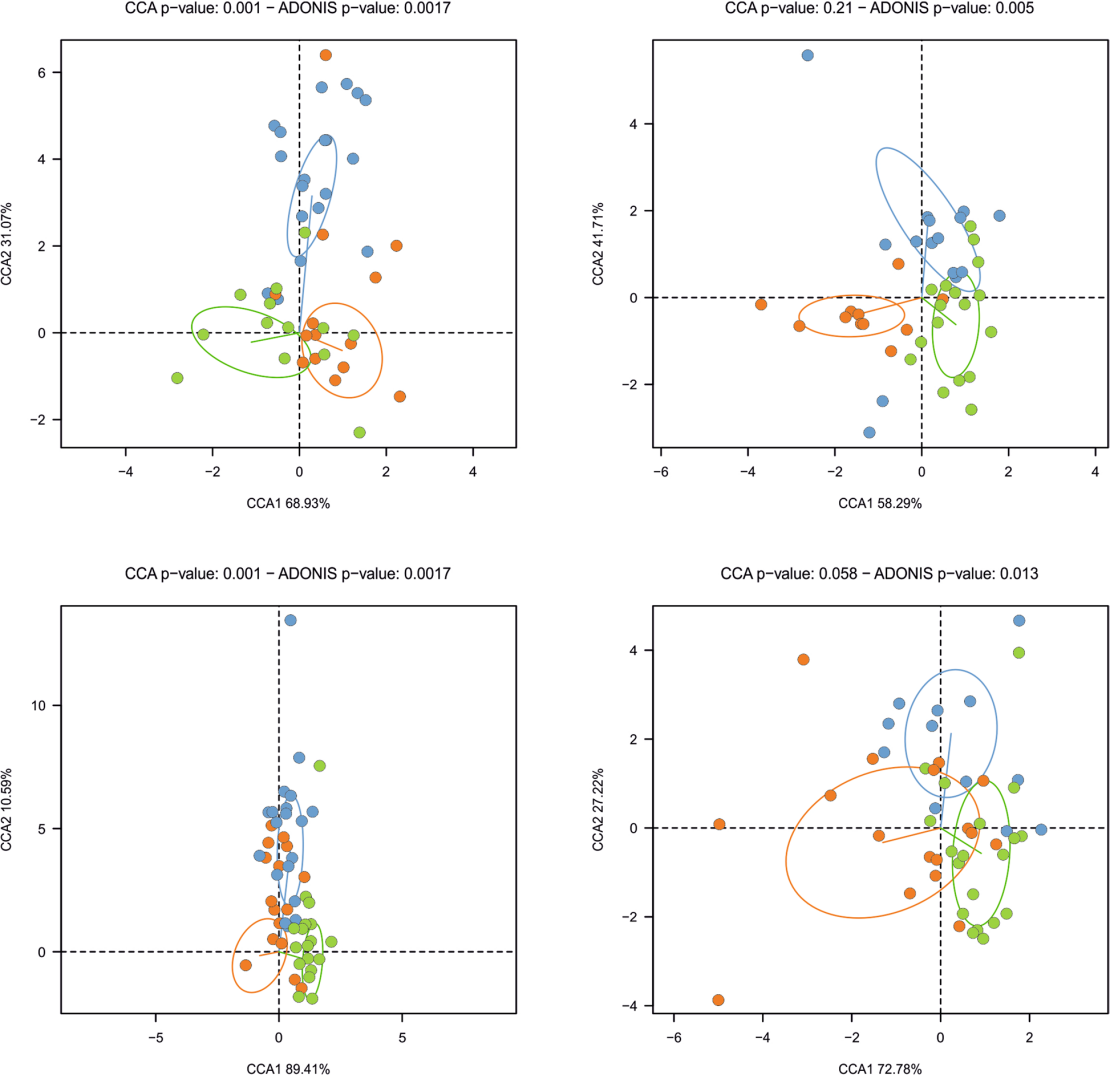
Effect of dentifrice treatment on taxonomic composition. Differences in abundance (MTG) and activity (MTT) of bacterial communities (species) between baseline (post-washout period), fluoride treatment, and fluoride + arginine treatment were studied using canonical correspondence analyses (CCA). The effect on caries-active (CA) and caries-free (CF) samples was studied separately. 95% confidence intervals were included

This is the first clinical study evaluating the change in the DNA and RNA functional profile of the oral microbiota after a prolonged use of different fluoride-containing dentifrice. The data show that there were differences observed between patients at baseline (after 1 week of brushing with the fluoride dentifrice) and at visit 2 (after 3 months of brushing with the same dentifrice). The MTG dataset was used to study the changes in the abundance of the different oral community members. In both CA (*p* = 0.001) and CF (*p* = 0.001) samples, a canonical correspondence analysis (CCA) showed a significant effect when the microbial composition was compared to baseline vs 3 months of brushing with the fluoride dentifrice (Fig. 3). Indeed, ecological shifts were observed for plaque taken from both the healthy and diseased (CA sites). This result illustrates that subjects that continued to brush with the same fluoride dentifrice for 3 months experienced a significant shift in bacterial composition, suggesting that “washout” periods in clinical trials, usually of a week in length, should probably be longer if the effect of toothbrushing on microbial composition is to be filtered out as a confounding factor. Interestingly, the changes observed in the active RNA-based microbiota as estimated by the MTT dataset were not significant (CA *p*-value = 0.21 and CF *p*-value = 0.058). Thus, although compositional changes were detected in the dental plaque bacterial communities, a comparable change in the gene expression was not observed. The microbiome compositional changes were likewise not specific to plaque taken from sound or caries active sites, indicating that the ecological shifts were observed in both health- and disease-associated communities.

Further analysis was completed to unravel the differences in bacterial species abundance among the different groups (Additional Fig. 5). After 3 months of brushing with the fluoride dentifrice, commensal species such as *Corynebacterium matruchotii*, *Actinomyces massiliensis*, or members of the family *Neisseriaceae* were increased in CA samples as were periodontal pathogens, including *Selenomonas*, *Prevotella*, and unclassified members of the family *Porphyromonadaceae* (Additional Table 2). For samples taken from CF subjects, *Prevotella* species were increased while *Streptococcus* spp., which are mainly saccharolytic, were reduced (Additional Table 3). Similarly, when 16S data were used for the same comparison, the abundance of potential periodontal pathogens such as *Leptotrichia*, *Tannerella*, *Fusobacterium*, *Selenomonas*, and *Saccharimonadaceace* were increased after 3 months of brushing with the fluoride dentifrice in CA and/or CF individuals (Additional Fig. 5).

When comparing the active microbiota, the number of species differentially represented in the MTT after 3 months of brushing with the fluoride dentifrice was smaller, supporting the CCA results (Additional Fig. 5 and Additional Tables 4 and 5). In CF patients, the increased levels of *Neisseria*, *Porphyromonas*, and *Prevotella* suggested that these members are not only more abundant but also more active after 3 months of brushing with the fluoride dentifrice compared to baseline. In addition, although the levels of unclassified *Streptococcus* were reduced, the activity of health-associated streptococci such as *S. oligofermentans* and *S. dentisani* was increased in the metatranscriptome.

### Effect of using a fluoride + arginine dentifrice on the microbiota

**Fig. 4:**
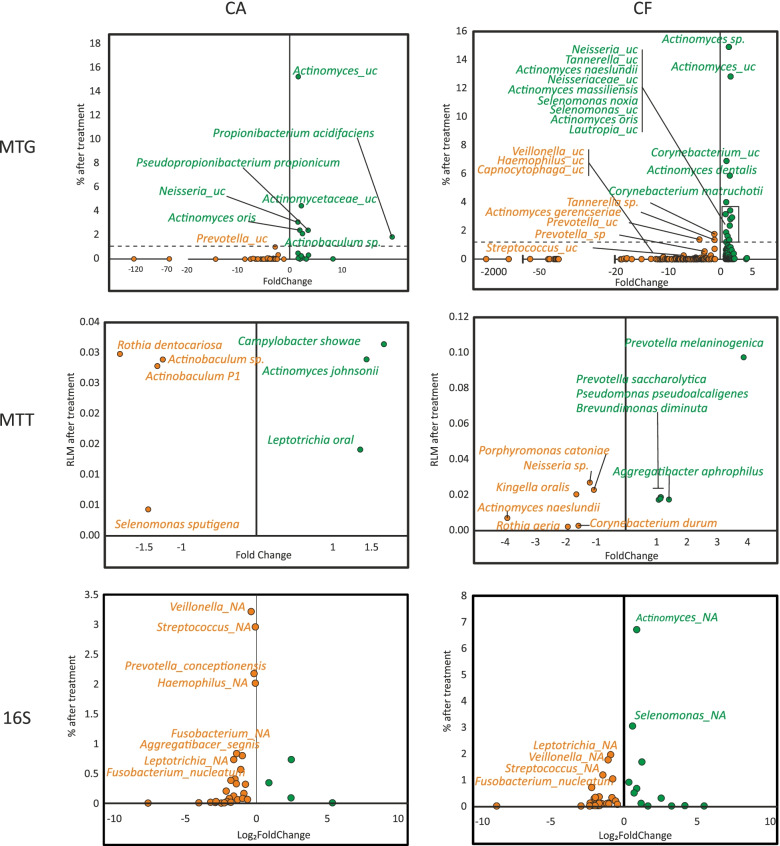
Changes in the levels of total and transcriptionally active bacterial species after fluoride + arginine treatments. Each dot represents a species that was significantly under- or over-represented in the 16S rRNA sequencing, metagenomic (MTG), or metatranscriptomic (MTT) dataset after a 6-month use of a fluoride + arginine dentifrice. The abundance (*y*-axis) and fold change (*x*-axis, %before/%after) are shown for each bacterium. Dots corresponding to species over-represented after treatment with a fluoride-containing dentifrice are shown in orange, and those in green represent bacteria over-represented after brushing with a Fl+Arg dentifrice. Species differentially represented in caries-active sites (CA) are shown on the left panels while those for caries-free individuals (CF) are on the right panels. The names of the species at > 1% abundance before or after the treatment are indicated

Similar to what we observed after we compared the microbiome of subjects brushing with a fluoride dentifrice at baseline vs 3 months, the microbiomes of these subjects at 3 months were also compared after using a Fl+Arg-containing dentifrice. After 6 months of use of a Fl+Arg dentifrice, the microbiome composition was further changed as revealed by the MTG and to a lesser extent by the MTT data (Fig. 4 and Additional Tables 6–9). Among those changes, the abundance of *Prevotella* species (which was increased when comparing the post washout vs 3 months fluoride dentifrice use time periods) was significantly reduced, and the abundance of *Actinomyces* and *Neisseria* increased in both CA and CF samples (Additional Tables 6 and 7). In addition, in CF individuals, there was a decrease in *Streptococci* and *Veillonella*, strongly indicating a further reduction in acidogenicity of the biofilm. Similarly, when 16S datasets were compared after using Fl+Arg dentifrice, *Actinomyces* was significantly increased and *Veillonella* and other proteolytic bacteria (*Fusobacterium*, *Prevotella*, or *Leptotrichia*) were reduced in both CA and CF individuals. Regarding the changes in active microbiota, only 7 and 12 species varied their activity significantly in CA and CF patients, respectively (Additional Tables 8 and 9). There was a reduction in the proportion of active species from several genera with high abundances in the MTG of CA patients (*Actinomyces*, *Corynebacterium*, or *Neisseria*).

Thus, although the microbial community changed from the baseline following the 3-month period of fluoride dentifrice use, brushing for 6 months with the Fl+Arg dentifrice resulted in further ecological shifts that significantly changed the composition and activity of dental plaque. In particular, compared to the 3 months MTG results, brushing with the Fl+Arg dentifrice promoted the increase of common oral commensal bacteria such as *Actinomyces*, *Corynebacterium*, or *Neisseria* and a decrease in sugar-fermenting streptococci. There was also a reduction in the abundance of *Veillonella*, which uses organic acids as the only source of carbon and therefore can be considered a biomarker for plaque acidogenicity [9, 33]*.* The arginine-induced changes in the microbiota composition were more apparent in plaque samples from caries-free individuals and were lower in CA sites (Additional Tables 6 and 7).

Finally, we separated the detected genera into two groups (arginolytic and non-arginolytic), based on the presence or absence of the *arcA* gene in the reference genomes, and compared the abundance/activity at baseline, after 3 months of brushing with a fluoride dentifrice, and after 6 months of brushing with a Fl+Arg dentifrice (Additional Fig. 6). As expected, the abundance of arginolytic bacteria was increased after the use of the Fl+Arg dentifrice in CF patients whereas there was no increase in the abundance of non-arginolytic bacteria. However, the abundances of some streptococci species (*S. cristatus*, *S. gordonii*, *S. sanguinis*, *S. intermedius*, *S. mitis*, or *S. oralis*), all well-known arginolytic bacteria, were reduced. This suggests that other arginolytic microorganisms were promoted. This would include *Actinomyces*, for which 18 different species were over-represented after the Fl+Arg treatment (Additional Table 7).

### General effect of using fluoride and Fl+Arg containing dentifrices on the functional profile

**Fig. 5:**
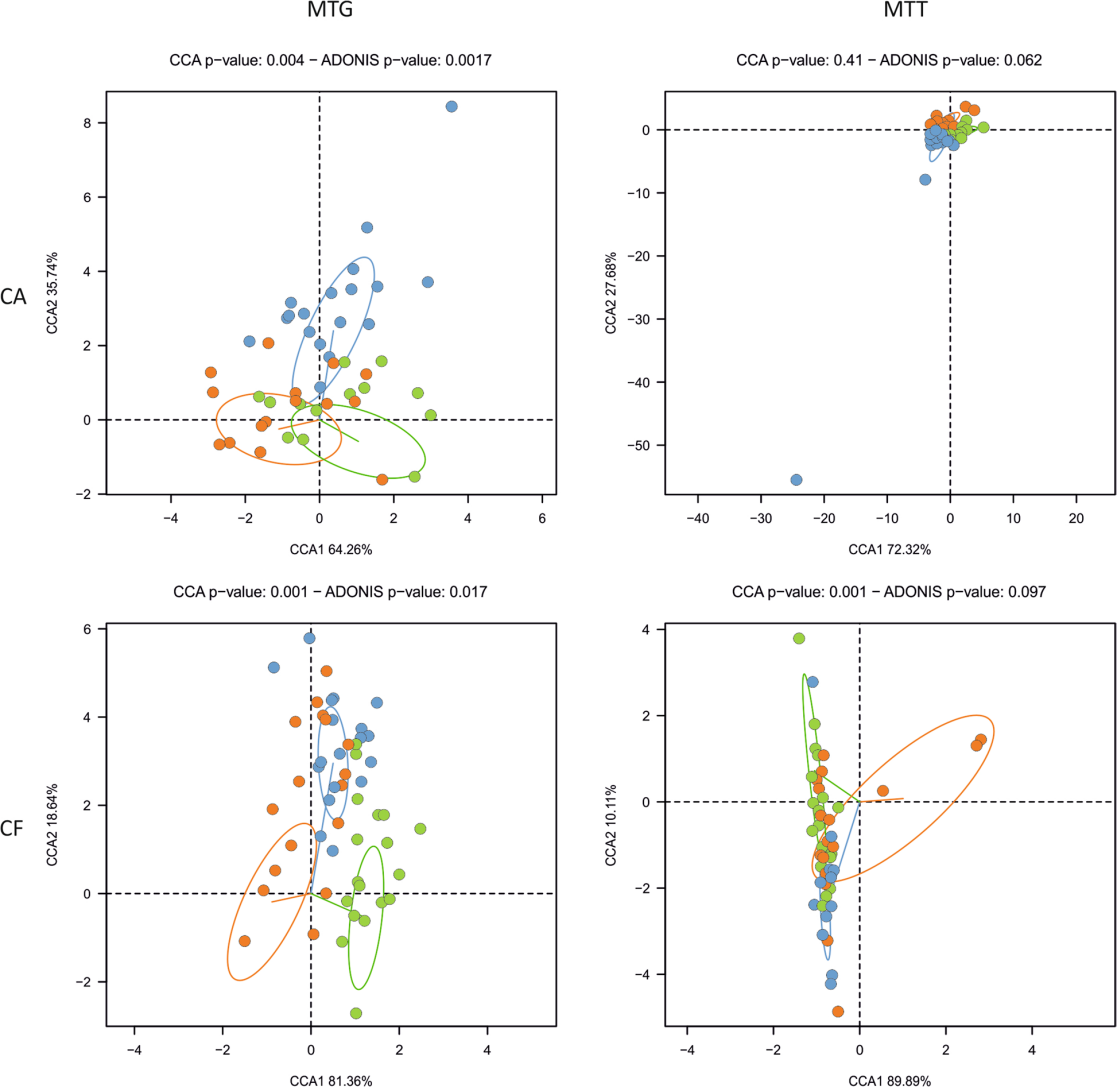
Effect of toothpaste treatment at the functional level. Differences in gene proportions (as inferred from the MTG dataset) and gene expression (as inferred from the MTT dataset) between baseline (post-washout period), fluoride treatment, and fluoride + arginine treatment were studied using canonical correspondence analyses (CCA). The effect on caries-active sites (CA) and caries-free (CF) patients was studied separately. 95% confidence intervals were included

Apart from providing information on taxonomic composition, the full potential of MTG and MTT data relies on the analysis of protein-coding genes and mRNA profiles, respectively. Thus, DNA and mRNA sequences were annotated, and the resulting functional profiles of the oral microbiota at baseline and after fluoride and Fl+Arg treatments were compared (Fig. 5). The results showed a significant effect of treatment when gene abundances were analyzed in both CA (*p* = 0.004) and CF (*p* = 0.001) samples. However, when the gene expression profiles were compared between the three sampling times, a large degree of overlapping was found, and a significant shift was only found for CF patients (*p* = 0.001), but not for CA samples (*p* = 0.41). As expected, there was little overlap between the MTG and MTT datasets in the number of genes over-represented (in the MTG data) or over-expressed (in the MTT data) for both CA and CF individuals (Additional Fig. 7).

According to DESeq2 analyses, there were over 400 genes differentially represented in the metagenomes of CA and CF samples after brushing for 3 months with the fluoride dentifrice (Additional Fig. 8, Additional Tables 10 and 11). We found several genes over-represented in those patients sampled after the 3 months brushing period with maximum similarity to a macrolide transport system ATP-binding/permease protein (*macB* and *msrA*) and macrolide efflux protein (*mef*). Given that this efflux protein has also been found to be over-expressed after fluoride exposure in in vitro oral biofilms [15], future work should test if the over-representation of any of the above genes could be a result of an increased frequency of exposure to fluoride dentifrices due to the 2×/day brushing requirement per-protocol instructions. This result could be an adaptation to export fluoride, due to its toxicity to most bacteria even at moderate concentrations. In order to compare the gene expression levels, the MTT data was used. The gene expression at baseline (i.e., after 1 week of use of fluoride dentifrice) compared to the MTT data obtained after 3 months of brushing showed 183 genes differentially expressed in CA samples and 309 in CF patients (Additional Fig. 9, Additional Tables 12 and 13).

### Effect of a fluoride + arginine dentifrice on the functional profile

The abundance of genes in the MTG and MTT datasets was compared between the fluoride and Fl+Arg groups to detect potential compositional and transcriptional changes induced by brushing with the Fl+Arg-containing dentifrice. Similar to what was observed after 3 months of brushing with the fluoride dentifrice, the microbial communities of CA samples (376 genes differentially abundant and 108 genes differentially expressed) were less affected by exposure to the Fl+Arg dentifrice than the CF samples (987 genes differentially abundant and 517 genes differentially expressed) (Additional Figs. 8 and 9, Additional Tables 14–17). This suggests that oral biofilms on caries lesions are more resilient to change by toothbrushing with fluoride and Fl+Arg dentifrices than biofilms on sound teeth surfaces from caries-free subjects.

**Fig. 6:**
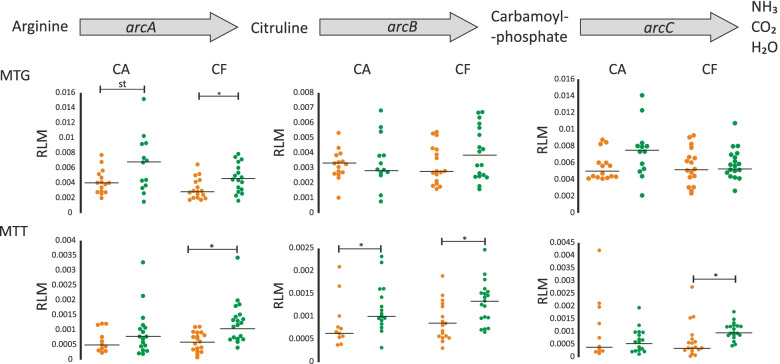
Changes in the arginolytic pathway gene content and expression after fluoride + arginine treatment. The degradation of arginine to NH_3_ and CO_2_ via the ADS pathway and the genes involved are represented at the top of the figure. The abundance of the genes (as inferred from the MTG dataset) in CA and CF patients before (orange) and after (green) the treatment is represented on the middle panels, while changes in the gene expression (as inferred from the MTT dataset) are shown on the bottom panels. *Statistically significant difference, *p* < 0.05. st, statistical trend, *p* < 0.1

In order to test whether arginine degradation was over-represented and/or over-expressed in oral biofilms after the Fl+Arg treatment, we compared the abundance and expression of the main genes involved in the arginine deiminase system (ADS), namely arginine deiminase (*arcA*), ornithine carbamoyl-transferase (*arcB*), and carbamate kinase (*arcC*). The MTG data showed that the abundance of *arcA* was significantly increased after using the arginine dentifrice in CF patients (*p* = 0.02) (Fig. 6). In addition, there was an increase in the expression (MTT data) of *arcA*, *arcB*, and *arcC* in CF patients (*arcA*, *p* = 0.002; *arcB*, *p* = 0.01; *arcC*, *p* = 1.64 × 10^−5^) and of *arcB* (*p* = 0.04) in CA samples (Fig. 6). This demonstrates that a 6-month exposure to brushing with a Fl+Arg dentifrice stimulates the arginolytic pathway, which is consistent with the observed higher arginine-derived ammonia production in patients using an arginine dentifrice in clinical studies [34, 35].

**Fig. 7:**
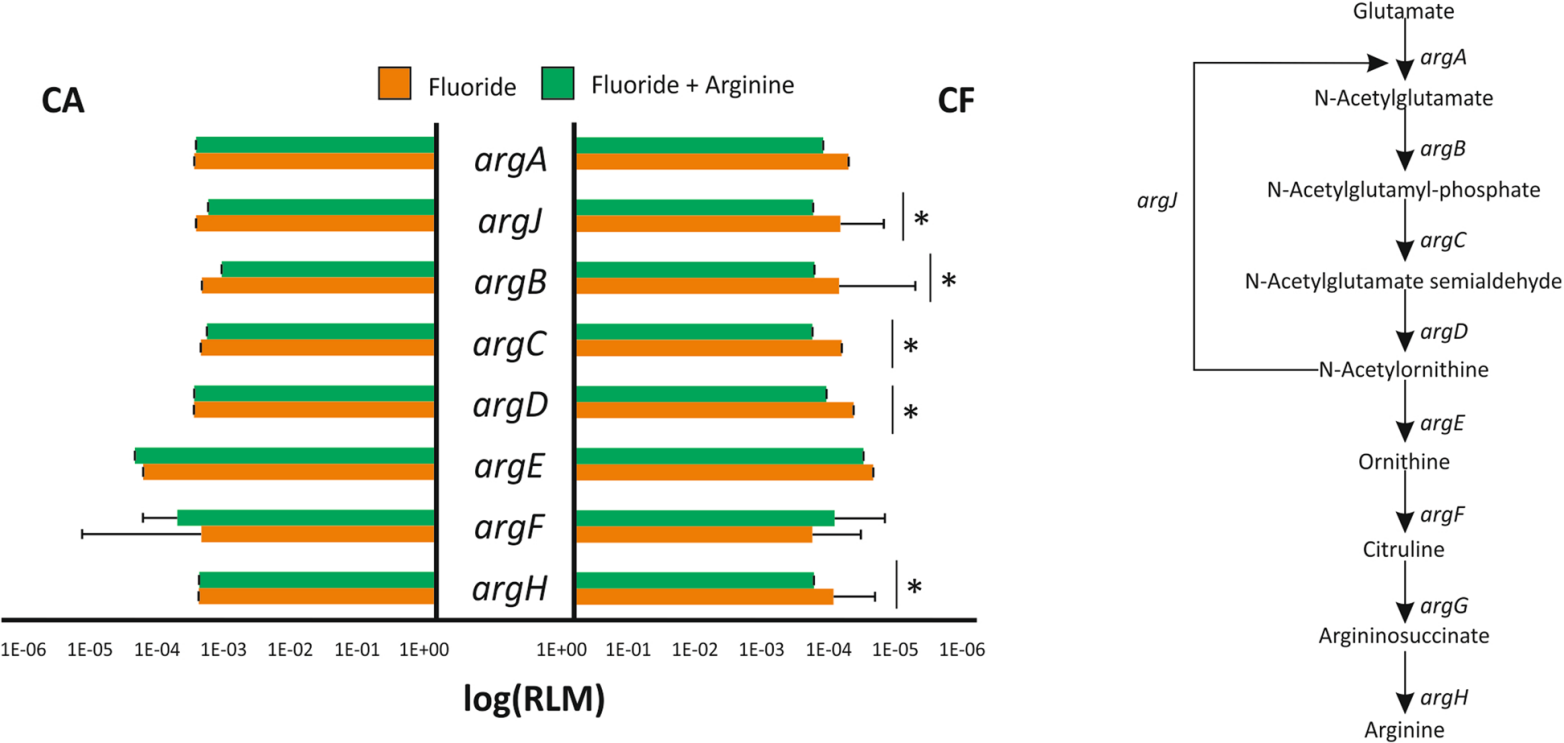
Changes in the arginine biosynthetic route after fluoride + arginine treatment. The abundance (logRLM) of each gene involved in the biosynthetic route is shown before and after treatment with fluoride + arginine. The pathway is shown on the right panel. *p < 0.05

Besides the arginolytic route, 5 out of 8 genes participating in the synthesis of arginine (*argBCDHJ*) were over-expressed for the CF patients after 3 months of brushing with the fluoride dentifrice in comparison with baseline and after brushing with the Fl+Arg dentifrice (Fig. 7). A repression of this pathway after arginine treatment is consistent with a reduced need to synthesize this amino acid due to its presence in the toothpaste and suggests that oral biofilms on sound enamel appear to be more efficient at producing innate arginine than caries-affected areas. An over-expression vs baseline could be a result of the subjects adapting to a different oral hygiene regimen of products and practices as a result of participating in the clinical study. The net impact is an improved oral environment resulting in a more enriched microbiota for the CF subjects who appear to be more biologically able to benefit.

In addition to arginine biosynthesis genes, 5 genes involved in arginine degradation into putrescine were over-represented and/or over-expressed when patients brushed for 3 months with the fluoride dentifrice vs baseline as well as the patients transitioned to using the Fl+Arg dentifrice: *aguA* (agmatine deiminase), *aguB* (N-carbamoylputrescine amidase), *speA* (arginine decarboxylase), *speB* (agmantinase), and *nspC* (carboxynorspermidine decarboxylase). Moreover, a subunit of the spermidine/putrescine complex (*potH*) was over-represented in CA and CF patients only after the 3 month brushing period. Therefore, brushing with the fluoride dentifrice favors the agmatine-mediated arginolytic pathway whereas brushing with the Fl+Arg dentifrice favors the standard pathway, which is known to be more efficient in buffering extracellular pH and also to be able to provide energy to ADS+ microorganisms through the production of ATP.

### Additional functional changes

In addition to those genes involved in arginine biosynthesis and degradation, there were other pathways that were differentially affected by fluoride or Fl+Arg treatments which could be relevant for oral health. For example, when comparing baseline vs fluoride, LPS biosynthesis-related genes were over-represented in CA and CF patients after the 3-month fluoride dentifrice brushing period (7 genes in CA and 6 genes in CF from which 4 were shared [*lpxA*, *lpxB*, *kdsA*, and *kdsD*]). Similarly, the same pathway was under-represented and under-expressed after brushing with Fl+Arg. In total, 57 out of 63 genes annotated as lipopolysaccharide biosynthesis were under-represented or repressed after Fl+Arg treatment in the MTG and MTT datasets when compared to brushing with fluoride dentifrice alone (Additional Tables 14–17). Therefore, this data suggests that LPS synthesis (or the bacteria with LPS-containing cell walls) may be repressed by brushing with a Fl+Arg dentifrice.

Regarding the genes over-represented after brushing for 3 months with the fluoride dentifrice vs baseline, 171 were detected in CA whereas 242 were detected in CF individuals (Additional Tables 10–13). Several genes were over-represented in both CA and CF patients such as those involved in fatty acid biosynthesis (*fas*, *fabG*, and *fabM*) and histidine metabolism. Histidine metabolism genes were over-expressed after brushing for 3 months with the fluoride dentifrice in comparison with the samples taken at baseline and were also over-represented and over-expressed after brushing with the Fl+Arg in comparison with those taken after 3 months of fluoride toothbrushing. This suggests that histidine metabolism was increased as subjects continued to brush with the fluoride dentifrice and increased even more after brushing with the Fl+Arg dentifrice. Thus, given the interconnection between the catabolic routes of different amino acids, arginine supplementation could also influence other amino acids’ metabolic pathways. This is also confirmed by the observation that glutamate ABC transporters (*gluA*, *gluB*, *gluC*, and *gluD*) were over-represented after brushing with the Fl+Arg dentifrice.

Interestingly, in CF patients, there were several amino acid-related transporters that were under-represented after the Fl+Arg treatment such as branched-chain amino acid transport system permease protein (*livH*, *livK*, and *livM*), spermidine/putrescine transport system ATP-binding protein (*potB*, *potC*, *potD*, and *potE*), and l-cysteine transport system substrate-binding protein (*tcyK*, *tcyM*, and *tcyN*). Given that the arginolytic pathway can lead to putrescine, this represents another example of how arginine metabolism can influence other connected pathways.

Regarding the genes codifying for phosphotransferase systems for sugars, only one component was over-represented in CA whereas 8 were over-represented in CF after the Fl+Arg treatment. At the transcriptional level, 4 and 6 genes were over-expressed after the treatment in CA and CF patients, respectively. Interestingly, fructose- and mannose-specific components (*manX* and *manZ*) were only over-expressed in CA patients whereas cellobiose-specific components (starch-related) were exclusively over-expressed in CF (*celA* and *celB*).

Other functions potentially relevant for biofilm formation are quorum sensing (QS) pathways. There were significant differences in both MTG and MTT datasets, with 13 QS genes over-represented or over-expressed after 3 months of brushing with the fluoride dentifrice and 18 QS genes over-represented or over-expressed after brushing with the Fl+Arg dentifrice (Additional Tables 10–17). Finally, analysis of the genes classified in the oxidative phosphorylation pathway suggested that there was a shift in the corresponding protein complexes expressed after dentifrice use in CF individuals. Specifically, six NADH:quinone oxidoreductase subunits (NQR) (*nuoGFMHDN*) were under-expressed whereas nine subunits of V/A-type H+/Na+-transporting ATPase (*ntpABCDEFHIK*) (8 with a fold change > 2) were over-expressed after the 6-month Fl+Arg treatment. Both complexes play a central role in cellular energy production, and the data suggest that arginine addition changed which system was preferably used for bacteria ATP production. At the transcriptional level, there was also a significant over-expression of ethanolamine utilization proteins (*eutLMHQBTS*) after the arginine treatment.

## Discussion

The current manuscript shows that the composition and functional profile of oral microbiota changed as a function of patients brushing with a fluoride or a Fl+Arg containing dentifrice. LPS biosynthesis, fatty acid biosynthesis, histidine metabolism, and macrolide transport were promoted during the 3-month fluoride dentifrice brushing period compared to baseline. Histidine metabolism was also promoted after brushing with the Fl+Arg dentifrice. The use of the Fl+Arg containing dentifrice, apart from stimulating the arginolytic pathway, favored the transport of several amino acids and glutamate, the PTS systems of several sugars, and ethanolamine utilization, as well as modified oxidative phosphorylation complexes. Although part of these changes could be due to physical plaque removal during toothbrushing, samples were collected 12 h after the last toothbrushing, and therefore, some of the specific pathways that showed metabolic shifts are congruent with an effect of the corresponding dentifrice’s active ingredients.

To our knowledge, this is the first report of a clinical study that has investigated the effect of dentifrice use on the oral microbiota at taxonomical and functional levels. It has to be kept in mind that it is difficult to establish the action of dentifrices’ active ingredients (especially fluoride) because participants may have been using a fluoride product before enrolling in the study and because volunteers participating in a clinical study normally improve their hygiene habits and skills during the study period. In order to ameliorate these confounding effects, clinical trials normally include a “washout period” of 1–2 weeks to homogenize oral hygiene product effects. Our study also included this standard homogenization period and was specially designed to test the effect of arginine by extending the period of brushing using a regular (i.e., no other actives) fluoride-containing dentifrice. The results obtained show a taxonomical and functional alteration in the oral microbiota when patients use a dentifrice with fluoride for 3 months compared to baseline, indicating that standard short washout periods may not be sufficient to control for the abovementioned confounding effects upon entering a clinical study. This long-term, cumulative effect of dentifrice use has also been observed after the use of oral hygiene products with stannous ions, although in this case, only taxonomic changes were studied within the salivary microbiome [36]. In the current work, plaque microbiome changes detected after 3 months of use of a fluoride dentifrice appeared to be similar in patients with and without active caries. On one hand, the abundance of the usual commensal species, including health-associated bacteria such as *Neisseria* was increased, suggesting that effective brushing with a fluoride-containing toothpaste benefits oral health. Lactate-utilizing *Veillonella* also decreased after 3 months of brushing with the fluoride dentifrice, suggesting that acid production was reduced, in agreement with perhaps improved oral hygiene and sugar fermentation inhibition induced by fluoride, as reported by several authors [12, 37]. On the other hand, potential periopathogenic species from the genus *Prevotella* and *Porphyromonas* increased, which could be a consequence of being favored under fluoride exposure or perhaps indicative of subjects using antibacterial products (i.e., zinc, stannous, CPC, or triclosan) prior the study start, whose effect could be eliminated after entering the study, when these kinds of products were not allowed. MTT data indicated that *Neisseria*, *Prevotella*, and *Porphyromonas* species were not only more abundant but also more active after 3 months of brushing with the fluoride dentifrice. Interestingly, even though 16S rRNA datasets showed differences at the compositional level when compared with MTG, the differences detected after the 3-month brushing period supported the MTG results. It has to be borne in mind that PCR of the 16S rRNA gene introduces a bias by under-amplifying bacteria with a sequence divergence at the primer binding sites [31], with a high G+C content [38] or with a low proportion in the sample [30]. Thus, the observed differences between the 16S rRNA and the metagenomic datasets underline the importance of performing whole DNA sequencing of human microbial communities to have a robust assessment of microbial composition and function. In this regard, the metagenomic dataset showed higher diversity than the 16S rRNA dataset, likely derived from an absence of PCR bias. A major role in this bias is a consequence of universal primers under-amplifying some bacterial groups, especially those that have a skewed G+C content, such as *Actinomyces*. Thus, our data suggest that previous 16S-based studies of dental plaque have consistently underestimated the levels of *Actinomyces* in dental plaque, as in our MTG dataset the levels of this organism are over 40% (Fig. 2 and Add. Fig. 2), and in our 16S dataset, they account for less than 10% of the total (Add. Fig. 3). This is part of the reason why a modest MTG sequencing depth allows covering a large part of the diversity. It must also be kept in mind that most of the detected bacterial species appear to be at very low proportions (shown in Fig. 2 in dark color as “others”). In fact, when we eliminate species detected at levels below 0.1% and repeat the rarefaction analysis, the estimated number of species goes down from 1700 to 131. As it can be seen in Fig. 2 and Add. Fig. 2, *Actinomyces* and *Corynebacterium* can account for over 50% of the metagenomic reads at baseline. Therefore, most sequencing reads will map to those abundant taxa, covering a large part of the abundant species, but a much larger coverage would be needed to detect changes in low-frequency microorganisms. It is also true that metagenomics is an expensive alternative and a high number of samples is not always affordable. In addition, the reduction in third-generation, single-molecule sequencing error rates suggest that in the near future the full sequence of the 16S rRNA gene will allow a more accurate taxonomic annotation.

Treatment with the dentifrice-containing fluoride and 1.5% arginine also induced changes at the taxonomical and functional levels. Firstly, the periodontitis-associated bacteria which had increased after 3 months of brushing with fluoride dentifrice (vs baseline) were significantly reduced, although other potential periodontal pathogens (i.e., *Selenomonas noxia*) were increased. The usual commensal members of the oral microbiota, including some health-associated organisms such as *Neisseria* and *Rothia* were favored after brushing with Fl+Arg. In addition, the MTG analysis of bacterial abundance after brushing with Fl+Arg suggested that arginine addition increased arginolytic bacteria. Thus, future work should study whether nitrate-reducing bacteria, which also produce ammonia [39] benefit from the presence of arginolytic bacteria or vice versa.

Our results generally agree with those from a previous study that compared the effects of an arginine-containing toothpaste in the microbiota of CA and CF individuals using 16S microarrays [32]. In their work, Nascimento et al. reported a decrease in *V. parvula*, *F. nucleatum*, a cluster of *Prevotella*, and *Slackia exigua* in CA patients whereas *Kingella oralis* and *Neisseria* sp. were reduced in CF individuals after treatment for 4 weeks with an arginine-containing dentifrice. Similarly, in our 16S rRNA gene datasets, the proportions of *F. nucleatum*, *F. periodonticum*, and *Prevotella nanceiensis* were reduced in CA individuals after the use of the arginine-containing toothpaste. Moreover, *F. nucleatum*, unclassified *Fusobacteria*, and unclassified *Veillonella* were reduced in the CF group. Other differences were not replicated in our study, although this could be influenced by the different treatment lengths (4 weeks vs 6 months) or the different methodologies (16S gene probe arrays vs 16S rRNA sequencing).

Interestingly, an increase of potential periopathogens observed after brushing with fluoride and Fl+Arg dentifrices has been previously reported [40]. In our study, the *fluoride* treatment by itself increased periopathogenic bacteria whereas brushing with the Fl+Arg dentifrice decreased these organisms but enhanced the growth of other potential periopathogens such as *Selenomonas* and *Treponema* species. This could be due to a reduction in acidogenicity of the biofilm at the expense of a more protein-rich environment, and the potential preference for proteolytic or alkalophilic communities should be further studied in the future. In this regard, it is generally accepted that a cariogenic plaque is normally saccharolytic and acidophilic whereas a periodontopathogenic plaque appears to be more proteolytic and alkalophilic, and therefore, the two microbial ecosystems leading to these two conditions could be favored by different external factors, ranging from salivary pH to dietary protein and carbohydrate intake [41, 42]. It is worth mentioning that most of the known periodontal pathogens are Gram-negative bacteria, which agrees with the increment in LPS biosynthesis-related gene expression observed when the subjects brushed with the control fluoride product. Nevertheless, it must be borne in mind that we did not collect data on the participants’ toothpaste or any information on oral hygiene regime use prior to the study. This implies that the increase in the aforementioned bacteria could be the result of a switch from an antibacterial toothpaste or mouthwash product (i.e., zinc, stannous, CPC, or triclosan) to the fluoride or Fl+Arg dentifrices in the current clinical study. Thus, future work should evaluate whether fluoride, whose efficacy in reducing caries risk is overwhelming [43], could in any way promote the growth of proteolytic bacteria. Lastly, it must be taken into consideration the fact that an accumulation of dental plaque is necessary for gum disease development. Therefore, even if toothbrushing with fluoride is shown in the future to promote periodontal pathogens, those would not be able to develop an inflammatory process because of the biofilm physical removal. In fact, Li et al. showed a reduction in the gingival index after 6 months using 8% arginine dentifrice [21].

These results might bring up a question on whether dentifrices should combine fluoride and arginine. Considering that arginine alone provides pH buffering capacity once the environment is acidic but does not initially prevent acidification of the plaque, and given that fluoride has been shown to inhibit several glucolytic pathways [12, 15], the combination of an active ingredient that represses sugar fermentation (fluoride) and another that stimulates pH buffering (arginine) would be complementary.

The MTG is well-known to represent the metabolic and genetic potential of a community and is expected to be more stable throughout the day whereas a metranscriptome provides a snapshot of the bacterial activity and gene expression at a specific sampling time [25, 27]. For this study, the plaque samples were collected 12 h after the last toothbrushing meal or drink (anything but water). We, therefore, hypothesize that the considerably more moderate changes in the MTT data are due to the collection of samples 12 h after eating or drinking. Changes in the bacterial activity would have been more apparent immediately after a meal or sugar challenge, and future studies should study the influence of these dentifrices on the gene expression at those relevant moments for caries development, as those transcriptional profiles could be informative of caries risk. It has also to be kept in mind that the number of samples in CA and CF groups for the MTT dataset was 18 for both groups at baseline, 15 vs 17 at 3 months, and 13 vs 18 at 6 months (Fig. 1). Therefore, it is possible that part of the observed differences between CA and CF sites could be due to the lower sample size in CA patients at those two time points. Regardless, we detected changes in some of the most active members of the bacterial community such as *Actinomyces* or *Corynebacterium* and also the mentioned changes in the arginolytic pathway.

Among the commensal microorganisms that increased after brushing with both dentifrices, *Corynebacterium matruchotti* could be relevant as it plays an instrumental role in biofilm architecture [44, 45]. *Actinomyces* species are commensal organisms in the oral cavity and are generally more active in dental plaque of healthy individuals in comparison with those with caries [46, 47]. However, some species in the *Actinomyces* genus can invade oral mucosa and enter the subcutaneous tissue causing actinomycosis [48], and others have been detected in higher concentrations in the subgingival tissue of caries-active individuals [33, 49, 50] or, in the case of *A. gerencseriae*, have been detected at higher activity levels in root caries lesions [51]. Interestingly, the abundance of *A. gerencseriae* was significantly reduced (*p* = 0.02) after brushing with the regular fluoride and Fl+Arg dentifrices. Finally, *Neisseria* and *Rothia*, which increased after the use of both dentifrices, are nitrate reducers generally associated with health in comparison with periodontitis patients [47, 52–54] and in comparison with individuals with dental caries [9] and halitosis [55].

Brushing with Fl+Arg dentifrices had an effect not only on arginine-related genes but also on many others, especially those related to amino acid metabolism. Compared to the 3-month samples, brushing with the Fl+Arg dentifrice changed the transport of several amino acids and glutamate, the PTS systems of several sugars, and promoted the utilization of ethanolamine as well as changed the oxidative phosphorylation complex.

**Fig. 8:**
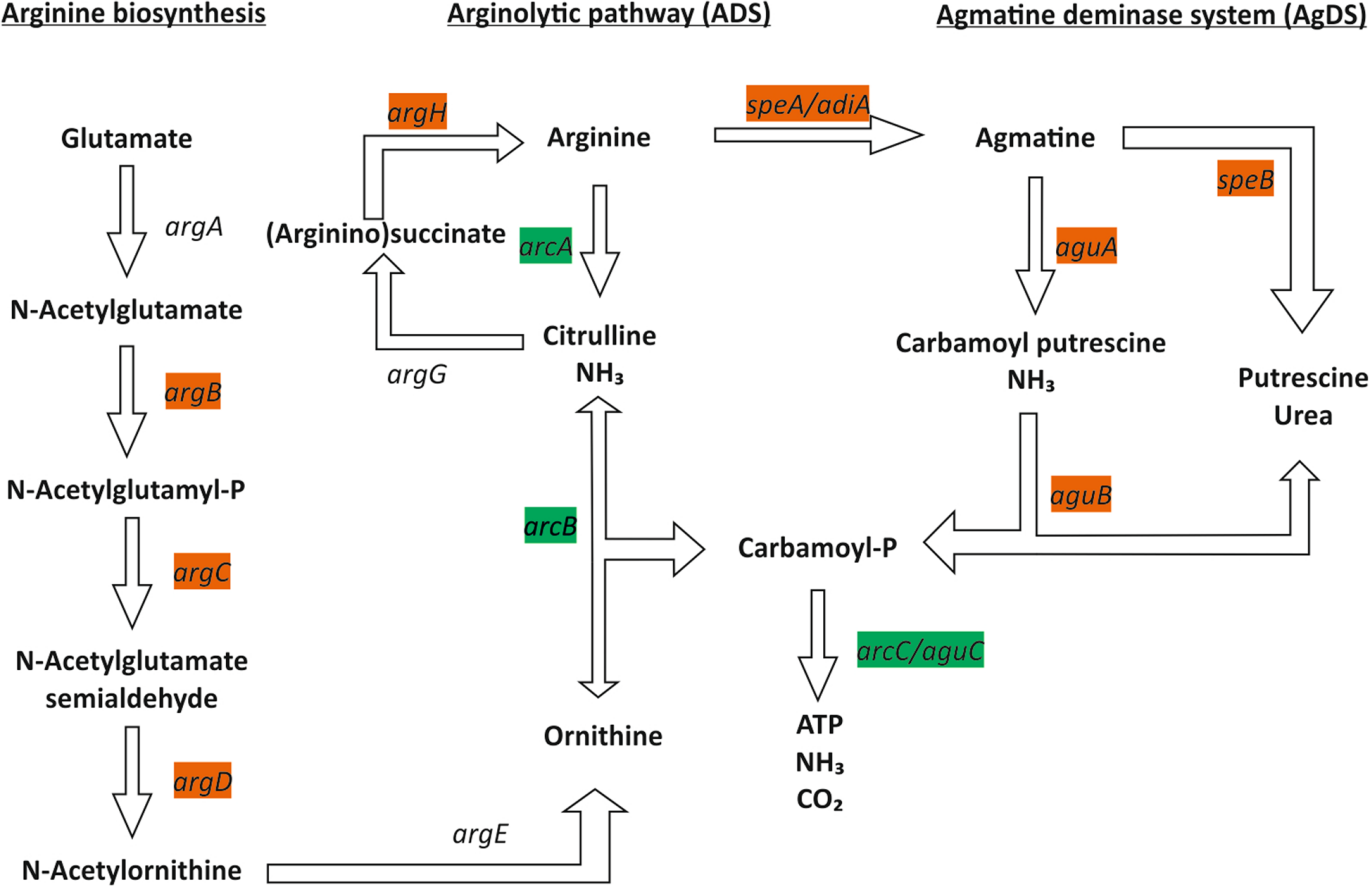
Effects of fluoride and fluoride + arginine on arginine metabolism. The enzymatic reactions and genes responsible for arginine biosynthesis, arginine degradation through citrulline (arginolytic pathway, ADS), or arginine conversion into agmatine and its further degradation (agmatine deiminase system, AgDS) are plotted. Those genes over-represented or over-expressed after fluoride treatment are colored in orange whereas those that were over-represented or over-expressed after fluoride + arginine treatment are colored in green

Regarding the ethanolamine utilization proteins, a similar pattern was found in pure cultures of *E. faecalis* after fluoride exposure [56], and the authors proposed that this bacterium reduced its carbohydrate metabolism and energy metabolism and increased ethanolamine utilization and amino acid metabolism to adapt and survive under fluoride exposure. Thus, it is unclear if the gene expression changes we observed in dental plaque samples are a consequence of the arginine supply or of the increased frequency of fluoride exposure. From those genes participating in the biosynthesis or catabolism of arginine, the MTG and MTT profiles suggest that the presence of arginine in the dentifrice repressed the biosynthesis of arginine and induced the degradation of arginine into ammonium and ATP (Fig. 8). Moreover, this arginolytic route through citrulline (arginine deiminase system (ADS)) was preferred over the alternative (agmatine deiminase system (AgDS)), which releases ammonia through agmatine/putrescine degradation. Previous reports on *S. mutans*, which has the AgDS pathway, have proven that ammonia production through the AgDS and the consequent alkalinization is smaller than ADS [56, 57]. Therefore, it is likely that the preferred ADS route observed after arginine treatment is the main reason why arginine deaminase activity and ammonia production have previously been found to be increased in clinical studies using arginine-containing dentifrices [32, 40, 58, 59] and confirms that pH buffering by the ADS is the metabolic pathway leading to decrease caries risk after prolonged arginine dentifrice use. In a recent metabolomic analysis, agmatine was found to be increased in dental plaque after a 3-month arginine treatment [24], which could be a consequence of accumulation due to reduced agmatine deiminase activity, and therefore, the effect of arginine on the AgDS pathway should be further explored. In addition, other complementary routes may also be taking place. For example, previous microbial studies on the effect of adding arginine into the dentifrice showed an over-expression in urease (*ureC*) after arginine treatment [58]. In our study, *ureC* was over-represented in CA patients after treatment with the Fl+Arg dentifrice which could contribute to pH buffering by urease-dependent ammonia production [60]. In addition, there was also a reduced lactate dehydrogenase expression after subjects brushing with the fluoride dentifrice, which could be responsible for a lower amount of lactate and therefore reduced acidogenicity. Previous clinical studies have also identified a reduction in lactic acid levels after the use of a Fl+Arg dentifrice [34, 35]. Finally, even though no samples from caries-free sites from CA patients were collected in the current work, further studies should evaluate the effect of dentifrices in these sites, which are clinically very relevant.

## Conclusions

Our data show that a prolonged toothbrush with either a fluoride- or Fl+Arg-containing dentifrice had an overall positive effect on the composition and functional profile of the dental plaque microbiota. The changes observed were beneficial for caries prevention since health-associated microbes appear to be favored by these treatments. Moreover, the addition of arginine into the dentifrice increased the abundance of arginolytic bacteria and the expression of genes involved in the arginolytic route, confirming that ammonia production through this pathway is activated and may contribute to reducing plaque acidogenicity. Altogether, the data indicate that the active ingredients in dentifrices, apart from having a chemical effect on tooth tissues, appear to be beneficial for caries prevention from a microbiological point of view, as a consequence of a taxonomic and functional shift associated with oral health. However, the potential increase of known periodontal pathogens must be further studied to determine the potential effect of dentifrices’ active ingredients on periodontal health. We acknowledge that the key to maintaining oral health is to minimize dysbiosis by following a healthy oral hygiene regimen. For some, this may mean using an oral hygiene product specially designed to manage caries or gum inflammation, and our data indicate that active ingredients in dentifrices, such as arginine, improve oral biofilm bacterial composition. We therefore propose that, in line with current views supporting the need of advancing in personalized dentistry tools [61], dentifrices should be developed that are specially designed for specific oral pathologies.

## Materials and methods

### Experimental design

An overview of the study is shown in Fig. 1. 53 patients (≥ 16 years old) were enrolled in a two-arm, longitudinal study, including 26 caries-active (CA) and 27 caries-free (CF) adults. Patients were considered CA if they had ≥ 3 active caries lesions (ICDAS scores 1–3), and CF if they had either ICDAS score 0 or ICDAS score of 1–3 where the lesion was inactive. During the course of the study, work relocation, inability to commit to the study appointments, switching dentifrices, antibiotics consumption, or smoking cessation tablets that cause dry mouth were considered as exclusion criteria. Being diagnosed with an advanced periodontal disease or other diseases that affects soft or hard oral tissues, having a medical condition which required premedication prior to dental visits, having orthodontic appliances that interfere with clinical assessments, having impaired salivary function, currently using drugs that can affect salivary flow, use of antibiotics 3 months prior the start of the study or arginine containing oral care products 3 months prior or during the study, being pregnant or breastfeeding, being in another clinical study 1 week prior to the start of the washout period or during this study, being allergic to common dentifrice ingredients or to amino acids, and immune-compromised individuals (HIV, AIDS, immune-suppressive drug therapy) were also considered as exclusion criteria. At the start of the study, subjects were instructed how to brush their teeth twice a day and had a washout period of 1 week using a dentifrice containing 1450-ppm fluoride as sodium monofluorophosphate (MFP) and an insoluble calcium compound. After 1 week, supragingival dental plaque was collected from either three active caries lesion sites in CA patients or 3 caries-free sites in caries-free patients by a sterile spoon excavator. Samples from the same participant were pooled in a tube containing RNAprotect (Qiagen, Hilden, Germany), thoroughly resuspended, and placed into dry ice to flash freeze. These samples correspond to the reference *baseline* (Fig. 1A). At this point, patients continued to use the same 1450-ppm MFP dentifrice with an insoluble calcium compound used during the washout period. After 3 months, another sample from the same sites was taken and processed by the same procedure (we refer to these 3-month samples as *fluoride*). Patients then received a dentifrice with 1.5% arginine and 1450-ppm fluoride as a sodium MFP and an insoluble calcium compound, which they used for an additional period of 6 months. After 6 months, a third set of samples was collected and termed Fl+Arg. Sampling at all time points was repeated a week after the initial visit to have a replicate of samples collected. Replicates were combined to obtain enough nucleic acid concentration to perform the metagenome and metatranscriptome analyses. The same CA or CF teeth were sampled at every collection time point. Patients were asked to visit the clinic at least 12 h after their last teeth brushing, meal, or drink (anything but water). All reported adverse effects during the study were not related to treatment.

Given that fluoride could have a long-term effect on bacterial communities, standard washout periods (1–2 weeks) may not be sufficient to eliminate fluoride as a confounding factor in testing the effect of arginine. Therefore, baseline samples were collected after the standard washout period of two weeks, and then a 3-month pretreatment with fluoride was selected as a more robust period for studying the effect of additional arginine, given that the arginine-containing toothpaste used also contained fluoride. The length (6 months) of the treatment with arginine-containing toothpaste was based on previous clinical trials where this period was sufficient to provide a clinical benefit. The study was registered on ClinicalTrials.gov with reference number NCT05138978.

### DNA and RNA extraction

RNA was extracted using the Ambion mirVana miRNA isolation kit (Thermo Fisher, Carlsbad, CA) and modified to include enzymatic digestion with lysozyme, lysostaphin, and mutanolysin plus bead beating. DNA was eluted from the organic phase of the RNA extraction and then ethanol precipitated.

### 16S rRNA gene sequencing

16S rRNA gene libraries were created as described by Caporaso et al. [62], employing primers that amplify the 16S rRNA gene variable region 4 (V4) region of all bacteria and archaea. A mock community [63] and no template controls were included to discard contamination and sequencing artifacts. Amplicons were quantified using the KAPA Library Quantification Kit for Illumina Platforms (KR0405, KAPABiosystems, Wilmington, MA), normalized, and pooled. Library quality was assessed by an Agilent Bioanalyzer chip then the library was prepared for sequencing on the Illumina MiSeq instrument using a MiSeq v3 Reagent kit (2 × 300 bp reads).

### DNA and cDNA sequencing

One hundred fourteen sample libraries were prepared using the Celero DNA-Seq kit (Tecan Genomics, Redwood City, CA) and sequenced on the Illumina NextSeq platform using 2 × 150 bp 550 High Output Kits. The remainder of the samples were not sequenced because of quality issues.

One hundred eleven samples were prepared as RNA-Seq libraries using the Ovation RNA SoLO kit (Tecan Genomics, Redwood City, CA). Eleven 150-bp paired-end libraries were sequenced on the Illumina MiSeq Platform during the optimization phase while the remaining libraries were sequenced as 150-bp paired-end libraries on the Illumina NextSeq Platform.

No rRNA depletion methods were used. According to our own experience and reported studies, the commercially available kits can introduce a significant bias in mRNA proportions [27]. Therefore, and considering that our samples do not have as much host RNA as other types of samples such as fecal or those coming directly from host tissues, no rRNA depletion kits were used. This implied a lower number of samples per run in order to obtain enough sequence coverage after bioinformatic rRNA removal.

### Bioinformatic analyses

For 16S rRNA analyses, we used the pipeline of dada2 [64] as previously described [65]. Briefly, reads filtered, end-trimmed, and denoised were merged, and later, chimeras and singletons were detected and removed. SILVA 16S rRNA non-redundant database was used for annotation [66].

For both metagenomic (MTG) and metatranscriptomic (MTT) reads, adapters (using *cutadapt*), short reads, reads with high percentage of ambiguous bases, and low mean quality reads were trimmed (using *prinseq*). Once cleaned, R1 and R2 metagenomic paired reads were joined using Flash [67]. R2 unaligned reads were discarded.

In both MTG and MTT samples, reads were mapped using bowtie2 (with the --very-sensitive option) to the human genome [68]. Aligned reads were removed from subsequent analysis. For the metatranscriptome, microbial ribosomal reads (rRNA treads) were detected and removed after aligning (bowtie2, --very-sensitive option) host-free reads to the SILVA database [66].

MTG reads were assembled into contigs using Megahit [68] and open reading frames (ORFs) were predicted in those contigs by PRODIGAL [69]. The number of reads (abundance) mapping each gene was calculated using R [70]. Kaiju [71] was used to assign each read to a taxon within the National Center for Biotechnology Information (NCBI) Reference Sequence (RefSeq) non-redundant database. The lowest common ancestor was computed in case of multiple hits. For reads that could not be annotated below a given taxonomic level (class, family, genus, etc.), we used the last taxon annotated as a reference and were described as “unclassified,” which is abbreviated in figures as “_uc.” These cases may derive from the limitations of short sequencing reads or due to the database being incomplete (for example, because the reads could belong to a yet uncultured species that could be present as a MAG [metagenome-assembled genome]). Finally, each gene was functionally annotated using HMMER against the databases TIGRFAM, KEGG, and PFAM.

For the annotation of the MTT dataset, reads were aligned with bowtie2 to a manually curated database. This database was based on the Human Oral Microbial Database (HOMD) [72] with the addition of genomes from bacteria isolated from the oral cavity that aligned a significant number of reads to our datasets or to publicly available oral MTG or MTT projects. The abundance of each gene was calculated using a manually created script with R (Additional Material and Methods). This abundance (number of reads) was normalized by the length of the gene (in bp) and the size of the dataset (in Megabp) giving rise to the RPKM metric (number of reads by gene length and by megabasepairs of mappable reads in the dataset) as done previously [73, 74]. The abundance of bacterial taxa was not affected by genome size, as suggested when the RPKM data were also normalized by genome size (Additional Fig. 10).

R language was used for statistical computing [70] to perform downstream analyses [70]. Rarefaction curves were performed with the minimum number of reads in a sample (10^5^ for MTG samples and 5 × 10^4^ for MTT samples). The Vegan library of R [75] was used for multivariant analysis (Adonis test—permutational multivariate analysis of variance using distance matrices [PERMANOVA]) and canonical correspondence analysis (CCA). The DESeq2 test was used for statistical comparisons, and false discovery rate (FDR) was used for multiple testing correction when MTG and MTT datasets were analyzed. Finally, 16S data were normalized and compared using the ANCOM-BC test [76], and diversity indexes were compared using the Wilcoxon test. Taxon with an abundance smaller than the closest abundance value to zero in the groups compared multiplied by four in less than 60% of samples was removed from the DESeq2 and ANCOM-BC analyses. All the analyses were unpaired.

## Supplementary Information

**Additional file 1: Additional Tables 1–17.** Additional tables are separated in sheets in a single excel file. The first line in each sheet contains the title of each additional table.

**Additional file 2: Additional Figure 1.** Relationship between sequencing effort and bacterial species and gene functions in human dental plaque. Rarefaction curves of all samples used for the current study when using metagenomic (A and B) or metatranscriptomic (C and D) data represent the number of species (left) and functions (right) detected relative to the number of sequence reads. Gene functions were calculated based on the KEGG database. Thresholds of 1x10^5^ reads/sample from the MTG and 5x10^4^ reads/sample for the MTT were used for annotation and subsequent analysis. Rarefaction curves at species level when samples were sequenced using 16S rRNA sequencing approach were also studied (E). Finally, the curves using metagenomics were also analyzed at species level when only species above 0.1% were considered (F).

**Additional file 3: Additional Figure 2.** Differences in bacterial abundance between metagenomic (MTG) and metatranscriptomic (MTT) data. The percentage of reads annotated to the top-20 most abundant genera is presented for both datasets in caries-active (CA) and caries-free (CF) samples. Baseline samples were collected after the washout period.

**Additional file 4: Additional Figure 3.** Differences in bacterial composition at genus level between datasets. (A) Bar graphs show the proportion (%) of the top-20 most abundant members of supragingival plaque microbiota at Baseline (post-washout period) and after Fluoride and Fluoride + Arginine treatments. At each time point, data are shown for the three available datasets (16S rRNA sequencing, shotgun metagenomics [MTG] and RNA sequencing [MTT]). CA and CF individuals are also differentiated. (B) PCA plot of samples from the CA and CF individuals at the three time points using 16S rRNA sequencing or MTG.

**Additional file 5: Additional Figure 4.** Number of species and genes detected (N), Chao1 and ACE richness indexes and Shannon diversity index for each study group. Data are shown for all three available datasets (16S rRNA sequencing, shotgun metagenomics [MTG] and RNA sequencing [MTT]). Baseline samples were collected after the washout period. Those comparisons which were significant (p<0,05) according to Wilcoxon test are indicated with *.

**Additional file 6: Additional Figure 5.** Changes in the levels of total and transcriptionally active species caused by toothbrushing with a fluoride toothpaste. Each dot represents a bacterial species that was significantly under- or over-represented in the 16S rRNA sequencing, metagenomic (MTG) or metatranscriptomic (MTT) datasets after 3 months of using a fluoride dentifrice. The abundance (y-axis) and the fold change (x-axis, %before/%after) is shown for each bacterial species. Dots corresponding to species over-represented at Baseline are colored in blue and those over-represented after treatment with fluoride are colored in orange. Species differentially represented in caries-active sites (CA) are at the left panels while those for caries-free individuals (CF) are at the right panels.

**Additional file 7: Additional Figure 6.** Presence of arginolytic and non-arginolytic bacteria at the different sample collection times. Bacterial species were separated into argynolytic and non-argynolytic (Non-*arcA*) according to the presence of the *arcA* gene. The abundance of each group was calculated at each time point. Data are separately shown for Caries-Active (A) and Caries-Free (B) individuals. Species abundance was normalized by the number of reads (R), by the length (L, in bp) of the gene and by the size of the dataset (in Megabasepairs).

**Additional file 8: Additional Figure 7.** Metagenomic and metatranscriptomic functional profiles correlation. The overlap between over-represented (MTG) and over-expressed (MTT) genes are represented as Venn diagrams. The genes that were over-represented and over-expressed are shown in the overlapping region for each comparison: Baseline vs Fluoride (left panel) and Fluoride vs Fluoride+Arg (right panel).

**Additional file 9: Additional Figure 8.** Changes in bacterial communities’ gene content and activity after Fluoride treatment. Each dot represents a gene that was significantly under- or over-represented in the metagenomics (MTG) or metatranscriptomic (MTT) datasets after 3-month use of fluoride dentifrice. The abundance/expression level in the MTG/MTT datasets (expressed as the number of reads normalized per gene length per Mpb of sequencing coverage) and the fold change (RLM before/RLM after) are shown for each gene. Dots corresponding to genes over-represented in baseline are colored in blue and those over-represented after Fluoride treatment are depicted in orange. Genes differentially represented in caries-active sites (CA) and Caries-free individuals (CF) are shown at the top and lower panels, respectively.

**Additional file 10: Additional Figure 9.** Changes in bacterial communities’ gene content and activity after Fluoride + Arginine treatment. Each dot represents a gene that was significantly under- or over-represented in the metagenomics (MTG) or metatranscriptomic (MTT) datasets after 6-month use of Fl+Arg dentifrice. The abundance/expression level in the MTG/MTT (expressed as the number of reads normalized per gene length per Mpb of sequencing coverage) and the fold change (RLM before/RLM after) are shown for each gene. Dots corresponding to genes over-represented after fluoride treatment are labeled in orange and those in green correspond to genes over-represented after Fl+Arg treatment. Genes differentially represented in Caries-Active sites (CA) and Caries-Free individuals (CF) are shown at the top and lower panels, respectively. AgDS, agmatine deiminase system; ADS, arginine deiminase system; NQR, NADH:quinone oxidoreductase; Na^+^ATPase, V/A-type H+/Na+-transporting ATPase; PTS, phosphotransferase system.

**Additional file 11: Additional Figure 10.** Effect of genome-size normalization on taxonomic composition. The abundance of the top-15 genera for the MTG dataset was normalized considering not only the size of the dataset but also by the corresponding genome size (Mbp).

**Additional file 12.** Additional Material and Methods.

## Data Availability

The datasets generated during the current study are available in the SRA repository with the accession number PRJNA712952.

## Abbreviations

CA: Caries-active
CF: Caries-free
MFP: Sodium monofluorophosphate
Fluoride: Treatment with dentifrice with 1450 ppm of fluoride
Fluoride+Arg: Treatment with dentifrice with 1450 ppm of fluoride and 1.5% of arginine
MTT: Metatranscriptome
MTG: Metagenome
