# Supplementary figures and images for: Functional changes in the oral microbiome after use of fluoride and arginine containing dentifrices: a metagenomic and metatranscriptomic study
Source: Microbiome. 2022 Sep 28;10:159. doi: 10.1186/s40168-022-01338-4 (PMC9520947; doi:10.1186/s40168-022-01338-4)

■ Baseline\_CA ■ Baseline\_CF ■ Fluoride\_CA ■ Fluoride\_CF ■ FI+Arg\_CA ■ FI+Arg\_CF

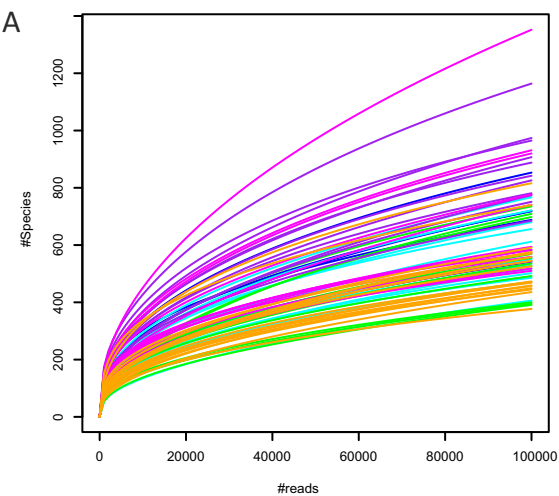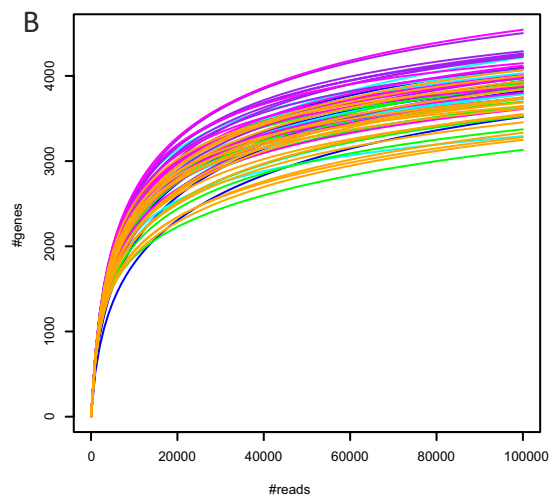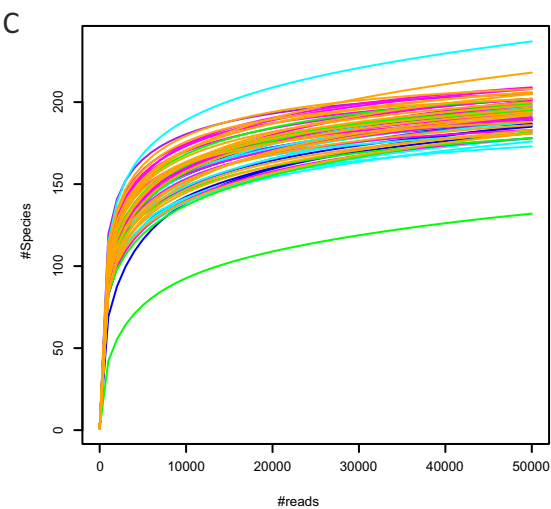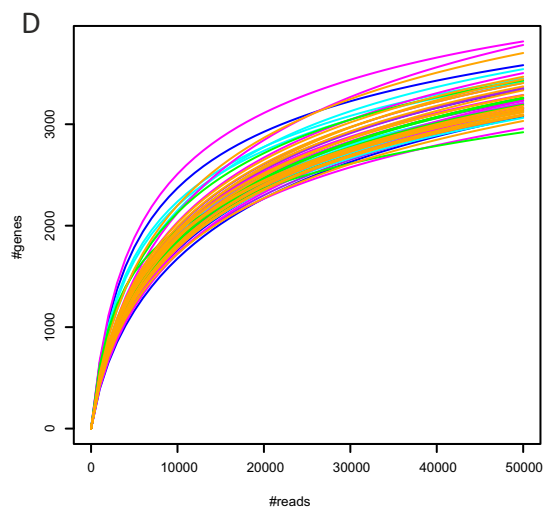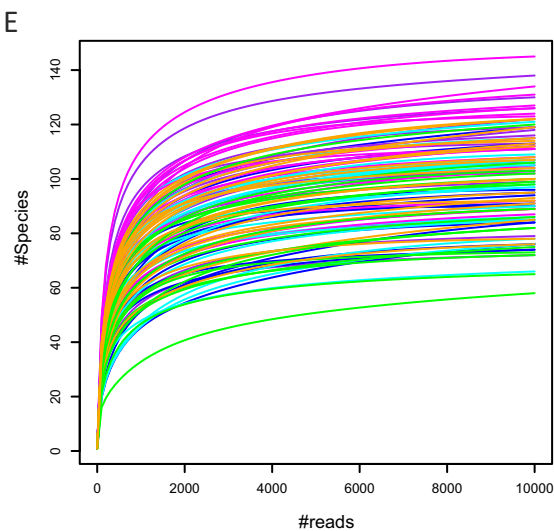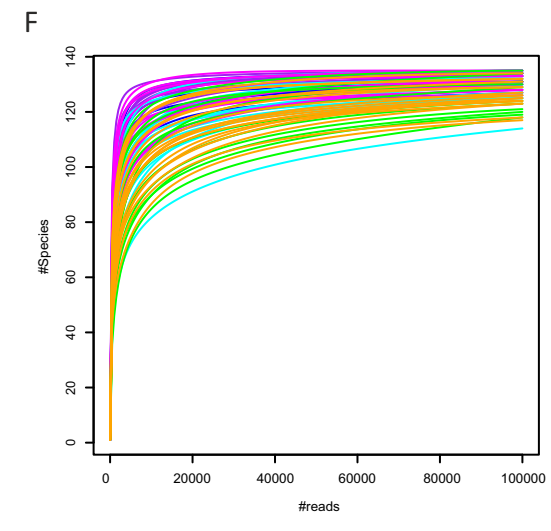

Supplement: Supplementary file 3 — Additional file 2: Additional Figure 1. Relationship between sequencing effort and bacterial species and gene functions in human dental plaque. Rarefaction curves of all samples used for the current study when using metagenomic (A and B) or metatranscriptomic (C and D) data represent the number of species (left) and functions (right) detected relative to the number of sequence reads. Gene functions were calculated based on the KEGG database. Thresholds of 1x10^5^ reads/sample from the MTG and 5x10^4^ reads/sample for the MTT were used for annotation and subsequent analysis. Rarefaction curves at species level when samples were sequenced using 16S rRNA sequencing approach were also studied (E). Finally, the curves using metagenomics were also analyzed at species level when only species above 0.1% were considered (F). [file 40168_2022_1338_MOESM2_ESM.pdf]

MTG

MTT

CA

CF

Baseline

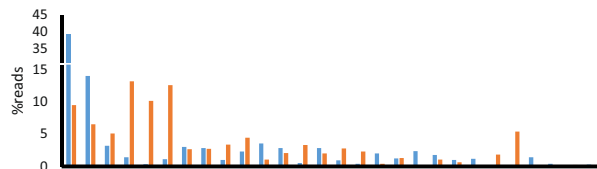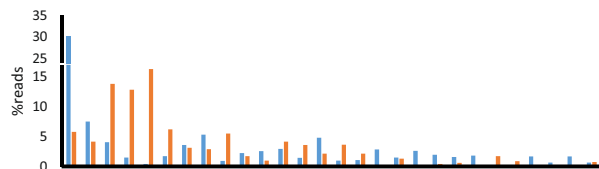

Fluoride

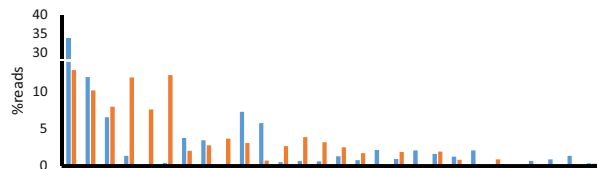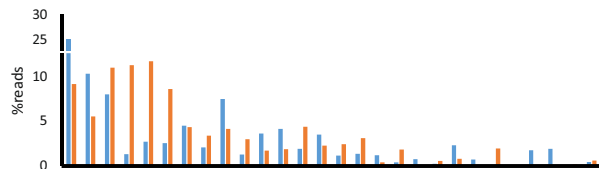

Fl+Arg

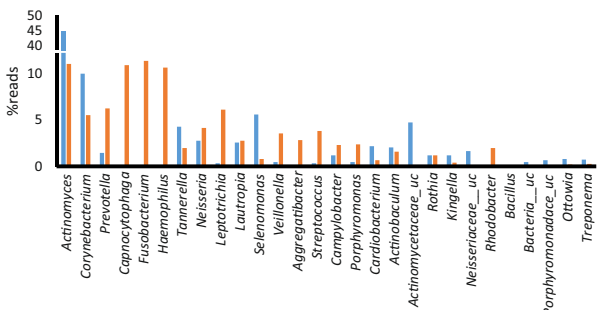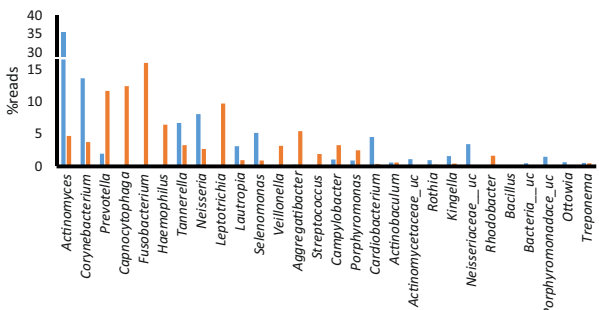

Supplement: Supplementary file 4 — Additional file 3: Additional Figure 2. Differences in bacterial abundance between metagenomic (MTG) and metatranscriptomic (MTT) data. The percentage of reads annotated to the top-20 most abundant genera is presented for both datasets in caries-active (CA) and caries-free (CF) samples. Baseline samples were collected after the washout period. [file 40168_2022_1338_MOESM3_ESM.pdf]

A

CA

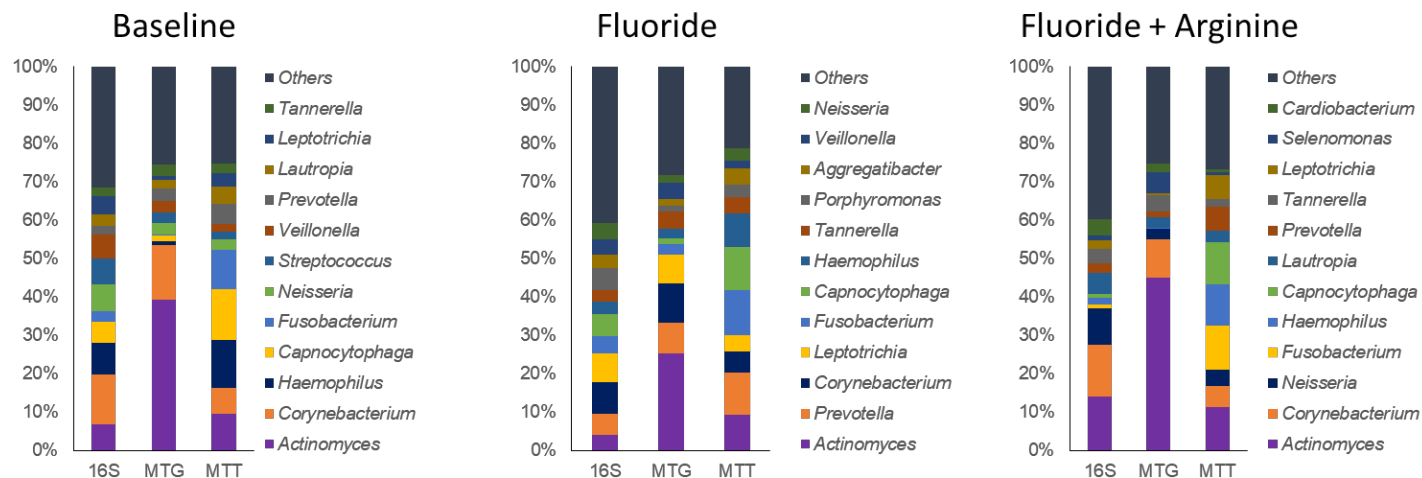

CF

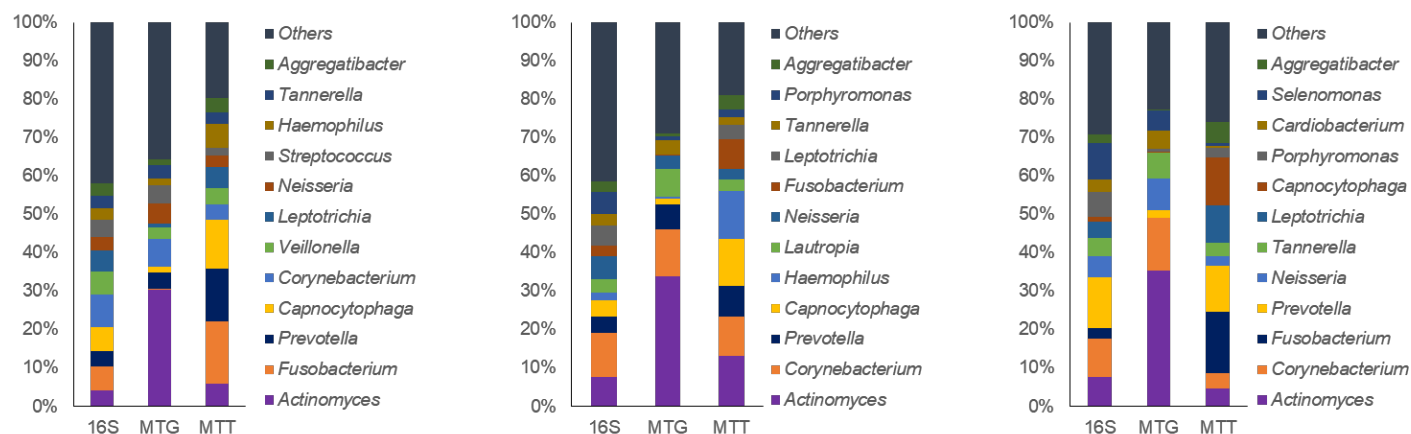

B

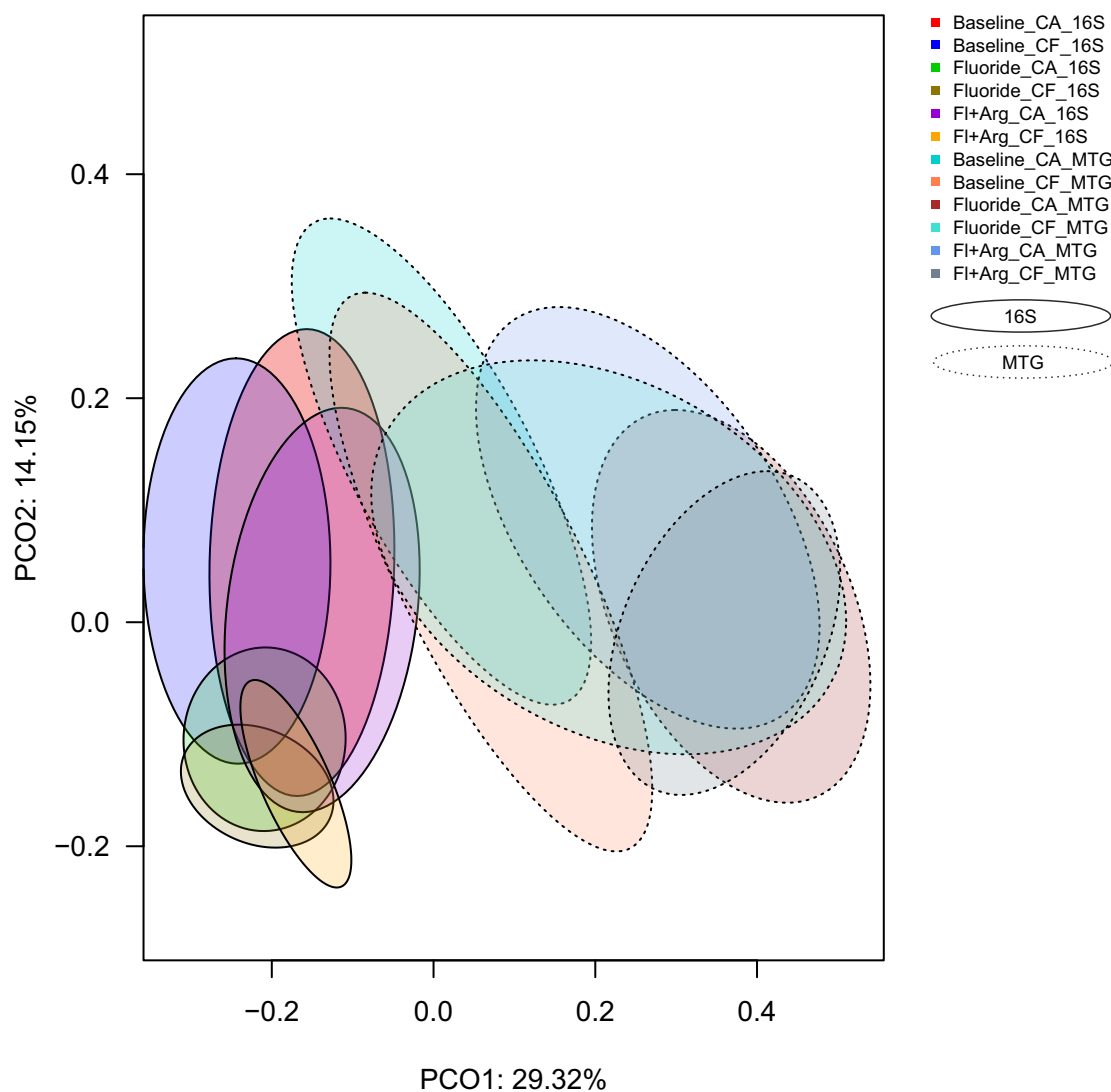

Supplement: Supplementary file 5 — Additional file 4: Additional Figure 3. Differences in bacterial composition at genus level between datasets. (A) Bar graphs show the proportion (%) of the top-20 most abundant members of supragingival plaque microbiota at Baseline (post-washout period) and after Fluoride and Fluoride + Arginine treatments. At each time point, data are shown for the three available datasets (16S rRNA sequencing, shotgun metagenomics [MTG] and RNA sequencing [MTT]). CA and CF individuals are also differentiated. (B) PCA plot of samples from the CA and CF individuals at the three time points using 16S rRNA sequencing or MTG. [file 40168_2022_1338_MOESM4_ESM.pdf]

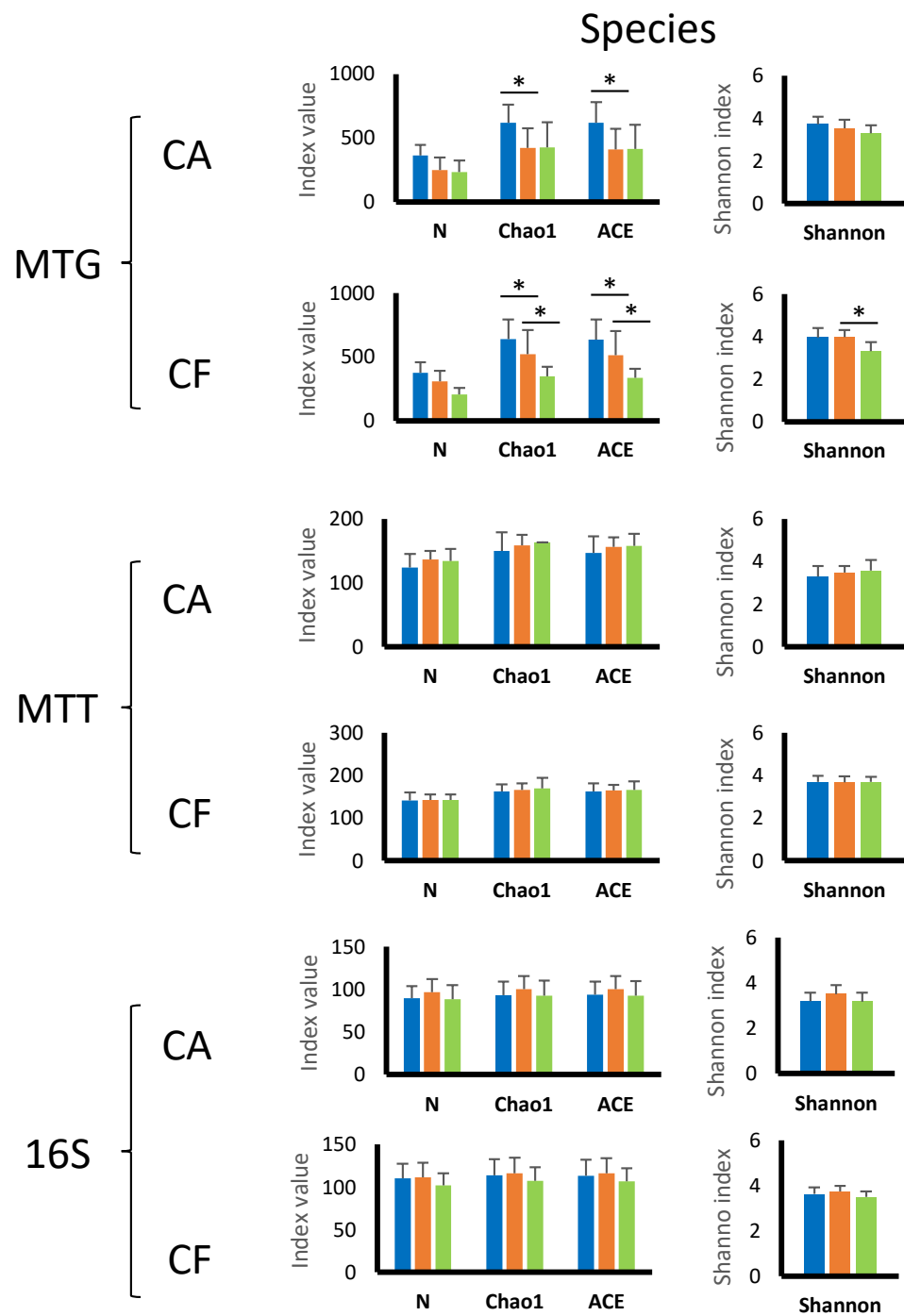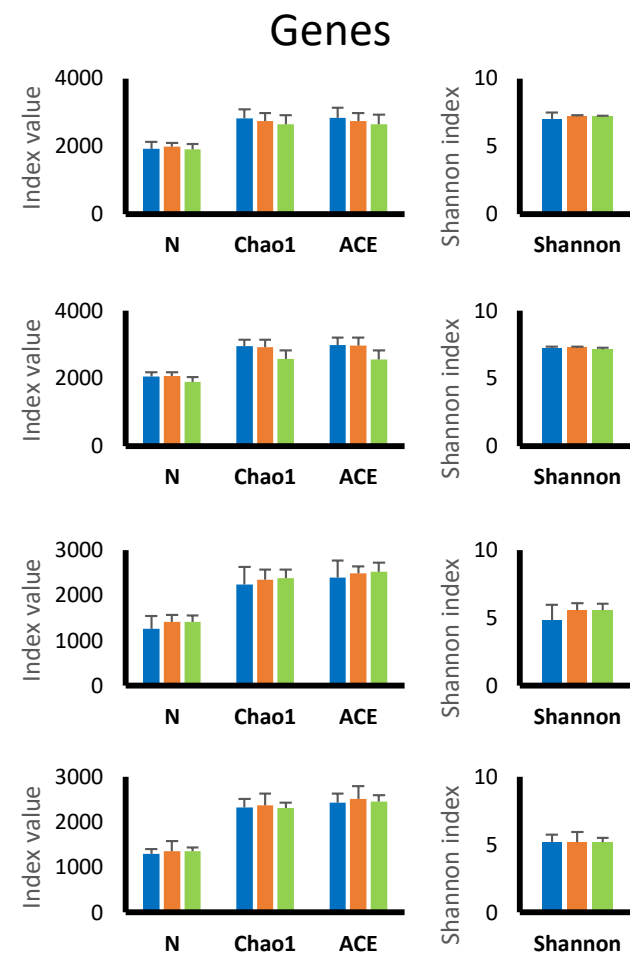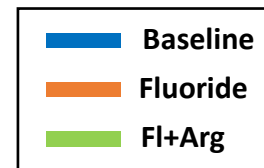

Supplement: Supplementary file 6 — Additional file 5: Additional Figure 4. Number of species and genes detected (N), Chao1 and ACE richness indexes and Shannon diversity index for each study group. Data are shown for all three available datasets (16S rRNA sequencing, shotgun metagenomics [MTG] and RNA sequencing [MTT]). Baseline samples were collected after the washout period. Those comparisons which were significant (p<0,05) according to Wilcoxon test are indicated with *. [file 40168_2022_1338_MOESM5_ESM.pdf]

CA

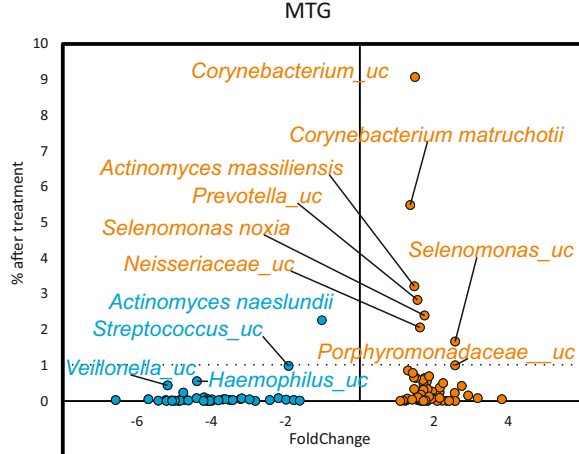

MTT

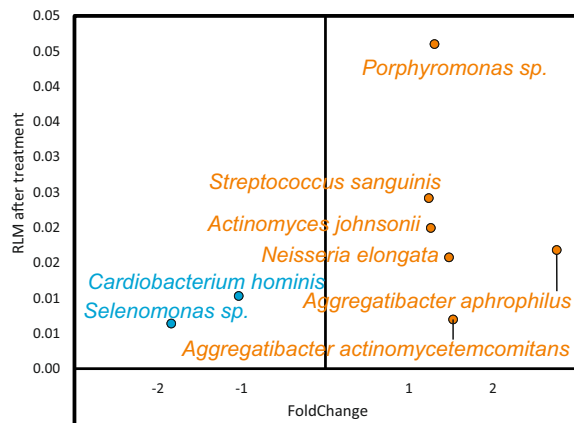

CF

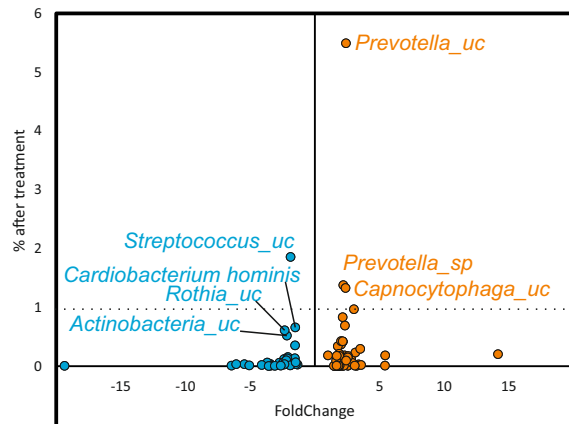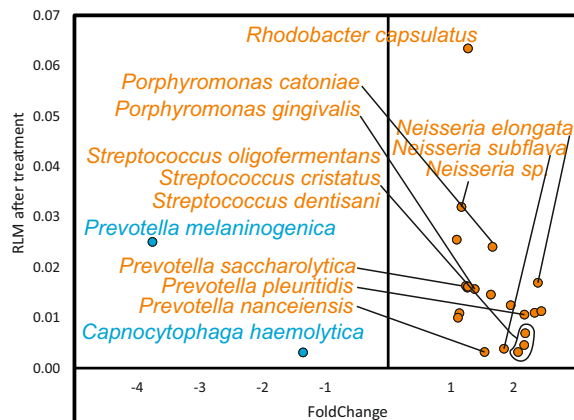

Supplement: Supplementary file 7 — Additional file 6: Additional Figure 5. Changes in the levels of total and transcriptionally active species caused by toothbrushing with a fluoride toothpaste. Each dot represents a bacterial species that was significantly under- or over-represented in the 16S rRNA sequencing, metagenomic (MTG) or metatranscriptomic (MTT) datasets after 3 months of using a fluoride dentifrice. The abundance (y-axis) and the fold change (x-axis, %before/%after) is shown for each bacterial species. Dots corresponding to species over-represented at Baseline are colored in blue and those over-represented after treatment with fluoride are colored in orange. Species differentially represented in caries-active sites (CA) are at the left panels while those for caries-free individuals (CF) are at the right panels. [file 40168_2022_1338_MOESM6_ESM.pdf]

**A**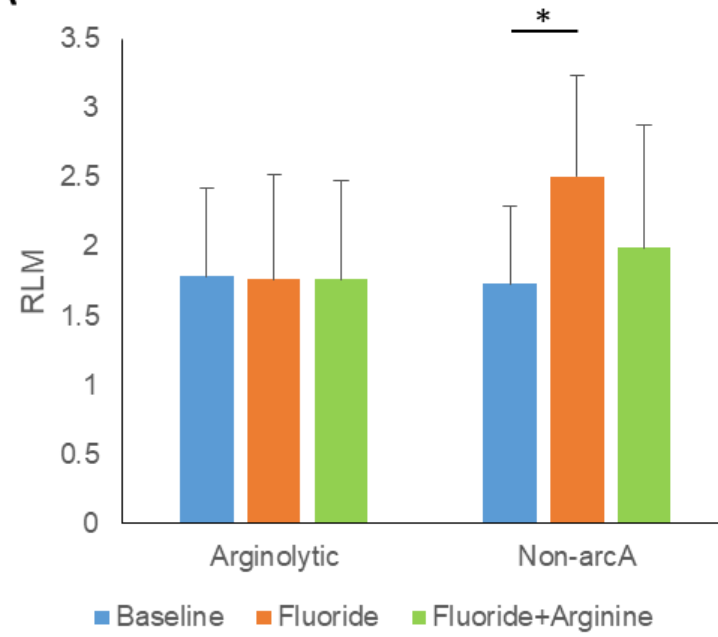**B**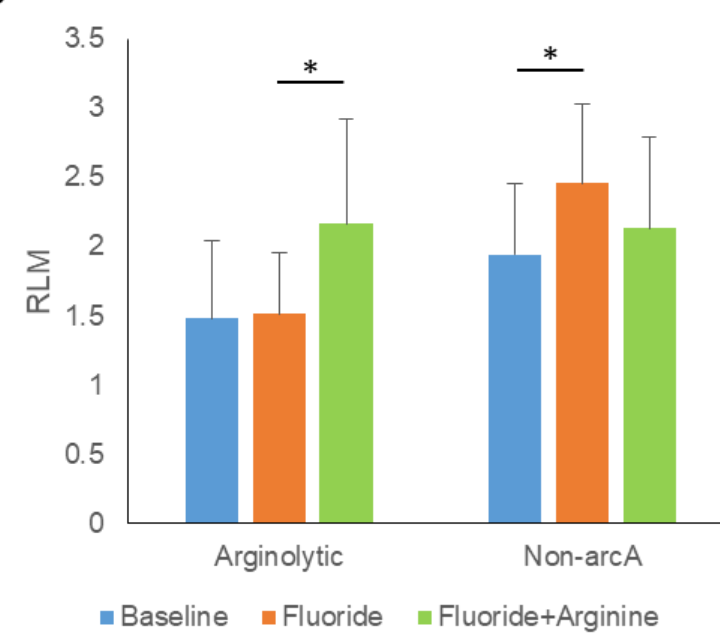

Supplement: Supplementary file 8 — Additional file 7: Additional Figure 6. Presence of arginolytic and non-arginolytic bacteria at the different sample collection times. Bacterial species were separated into argynolytic and non-argynolytic (Non-arcA) according to the presence of the arcA gene. The abundance of each group was calculated at each time point. Data are separately shown for Caries-Active (A) and Caries-Free (B) individuals. Species abundance was normalized by the number of reads (R), by the length (L, in bp) of the gene and by the size of the dataset (in Megabasepairs). [file 40168_2022_1338_MOESM7_ESM.pdf]

### Baseline vs Fluoride

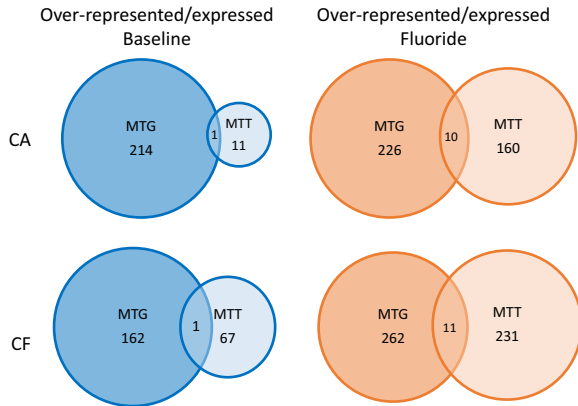

### Fluoride vs FI + Arg

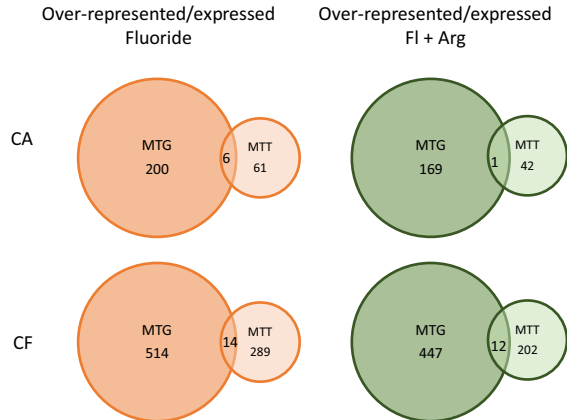

Supplement: Supplementary file 9 — Additional file 8: Additional Figure 7. Metagenomic and metatranscriptomic functional profiles correlation. The overlap between over-represented (MTG) and over-expressed (MTT) genes are represented as Venn diagrams. The genes that were over-represented and over-expressed are shown in the overlapping region for each comparison: Baseline vs Fluoride (left panel) and Fluoride vs Fluoride+Arg (right panel). [file 40168_2022_1338_MOESM8_ESM.pdf]

CA

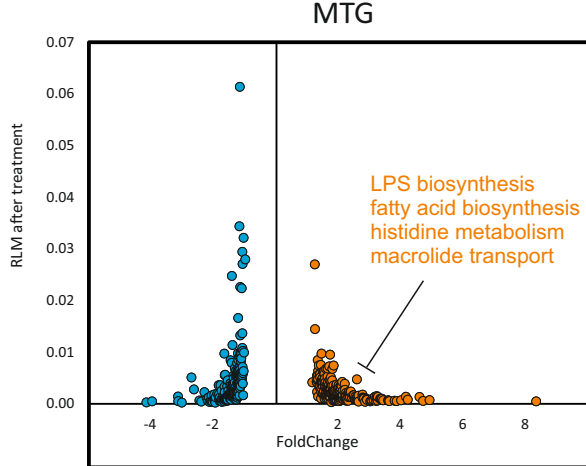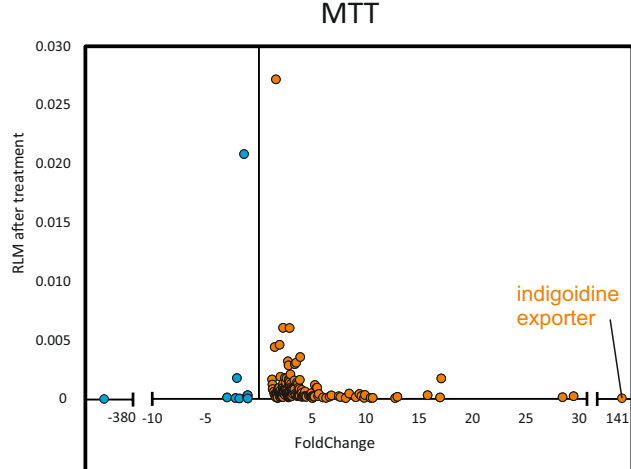

CF

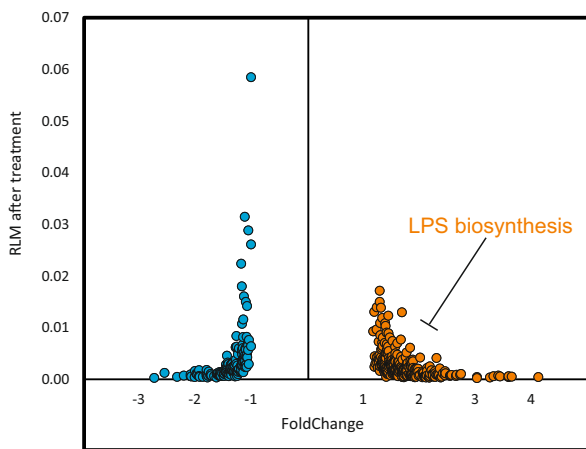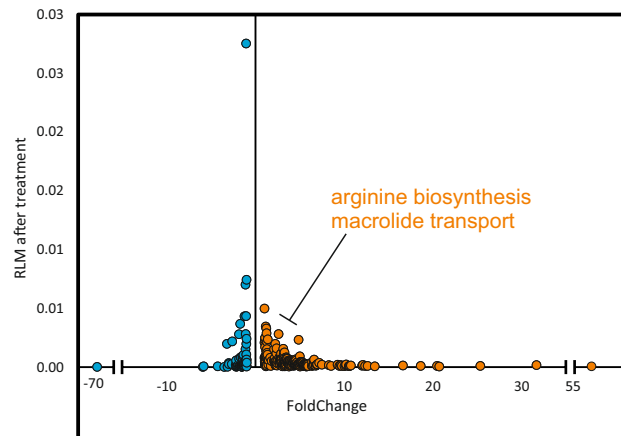

Supplement: Supplementary file 10 — Additional file 9: Additional Figure 8. Changes in bacterial communities’ gene content and activity after Fluoride treatment. Each dot represents a gene that was significantly under- or over-represented in the metagenomics (MTG) or metatranscriptomic (MTT) datasets after 3-month use of fluoride dentifrice. The abundance/expression level in the MTG/MTT datasets (expressed as the number of reads normalized per gene length per Mpb of sequencing coverage) and the fold change (RLM before/RLM after) are shown for each gene. Dots corresponding to genes over-represented in baseline are colored in blue and those over-represented after Fluoride treatment are depicted in orange. Genes differentially represented in caries-active sites (CA) and Caries-free individuals (CF) are shown at the top and lower panels, respectively. [file 40168_2022_1338_MOESM9_ESM.pdf]

CA

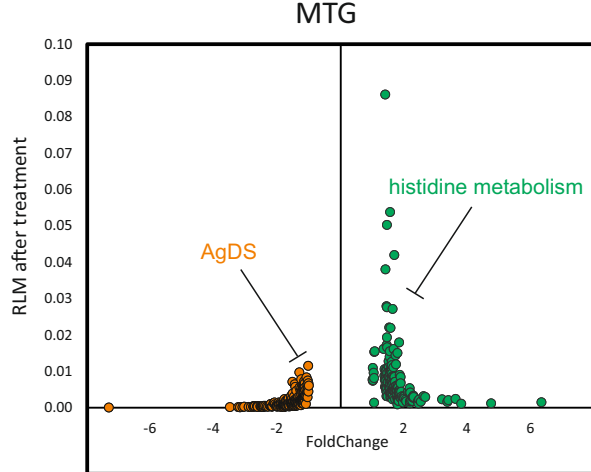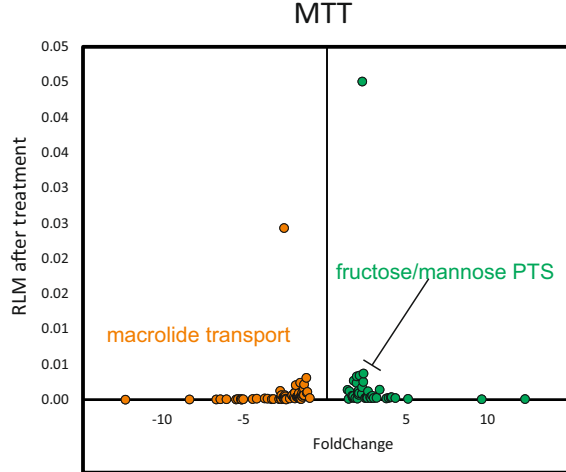

CF

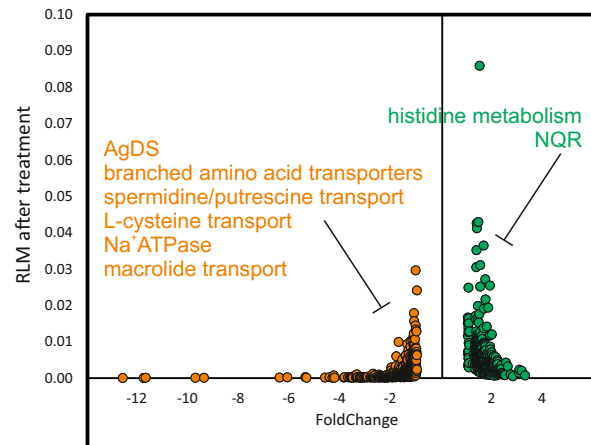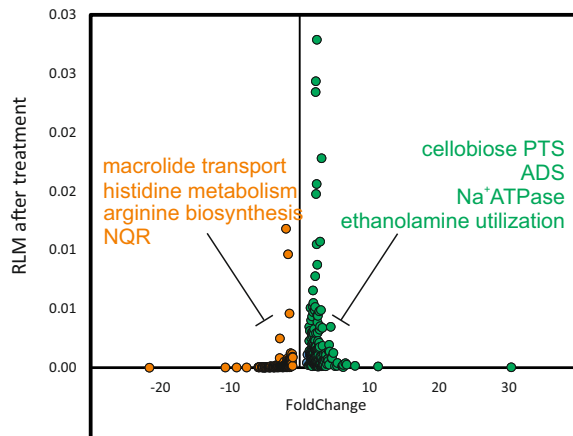

Supplement: Supplementary file 11 — Additional file 10: Additional Figure 9. Changes in bacterial communities’ gene content and activity after Fluoride + Arginine treatment. Each dot represents a gene that was significantly under- or over-represented in the metagenomics (MTG) or metatranscriptomic (MTT) datasets after 6-month use of Fl+Arg dentifrice. The abundance/expression level in the MTG/MTT (expressed as the number of reads normalized per gene length per Mpb of sequencing coverage) and the fold change (RLM before/RLM after) are shown for each gene. Dots corresponding to genes over-represented after fluoride treatment are labeled in orange and those in green correspond to genes over-represented after Fl+Arg treatment. Genes differentially represented in Caries-Active sites (CA) and Caries-Free individuals (CF) are shown at the top and lower panels, respectively. AgDS, agmatine deiminase system; ADS, arginine deiminase system; NQR, NADH:quinone oxidoreductase; Na+ATPase, V/A-type H+/Na+-transporting ATPase; PTS, phosphotransferase system. [file 40168_2022_1338_MOESM10_ESM.pdf]

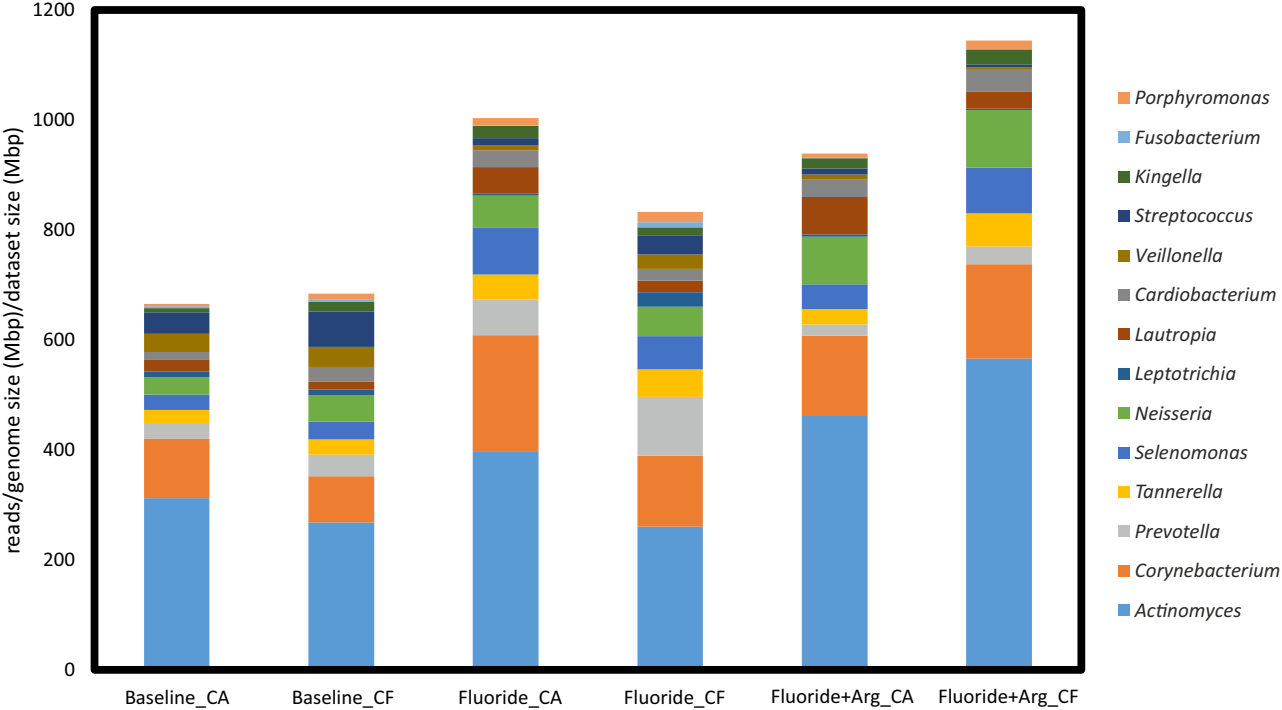

Supplement: Supplementary file 12 — Additional file 11: Additional Figure 10. Effect of genome-size normalization on taxonomic composition. The abundance of the top-15 genera for the MTG dataset was normalized considering not only the size of the dataset but also by the corresponding genome size (Mbp). [file 40168_2022_1338_MOESM11_ESM.pdf]
