# Supplementary material for: Functional changes in the oral microbiome after use of fluoride and arginine containing dentifrices: a metagenomic and metatranscriptomic study
Source: Microbiome. 2022 Sep 28;10:159. doi: 10.1186/s40168-022-01338-4 (PMC9520947; doi:10.1186/s40168-022-01338-4)
Supplement: Supplementary file 13 — Additional file 12. Additional Material and Methods. [file 40168_2022_1338_MOESM12_ESM.docx]

#!/usr/bin/env Rscript

suppressPackageStartupMessages(library(GenomicAlignments))

suppressPackageStartupMessages(require(parallel))

parameters <- commandArgs(trailingOnly=TRUE)

WORKDIR <- as.character(parameters[1])

SAMPLESFILE <- as.character(parameters[2])

GENESCOORDSFILE <- as.character(parameters[3])

NCPU <- as.integer (parameters[4])

SAMPLES <- readLines(SAMPLESFILE)

df.genes <- read.table(file=GENESCOORDSFILE, sep="\t", header=TRUE,

stringsAsFactors=FALSE, quote="", check.names=FALSE,

comment.char="", colClasses="character")

rownames(df.genes) <- df.genes$GENE

df.genes$START <- as.integer(df.genes$START)

df.genes$STOP <- as.integer(df.genes$STOP)

df.genes$GENE_LENGTH <- df.genes$STOP - df.genes$START + 1

## Gene abundance in each sample

for(SAMPLE in SAMPLES)

{

print(SAMPLE)

df.alns <- read.table(file=file.path(WORKDIR, SAMPLE, paste(SAMPLE,".sam",sep="")),

sep="\t", header=TRUE, stringsAsFactors=FALSE, quote="",

check.names=FALSE, comment.char="")

i <- which(bitwAnd(df.alns$FLAG,16)==16)

df.alns$STRAND <- "+"

df.alns$STRAND[i] <- "-"

## Gene abundance in each contig

genes.counts <- unlist(mclapply(unique(df.alns$REFERENCE_NAME), mc.preschedule=TRUE, mc.cores=NCPU, FUN=function(CONTIG)

{

df.genes.contig <- df.genes[df.genes$CONTIG == CONTIG,]

df.alns.contig <- df.alns[df.alns$REFERENCE_NAME == CONTIG,]

genes.ranges <- IRanges(start=df.genes.contig$START , end=df.genes.contig$STOP , names=df.genes.contig$GENE)

alns.ranges <- IRanges(start=df.alns.contig$REFERENCE_START, end=df.alns.contig$REFERENCE_STOP, names=df.alns.contig$QUERY_NAME)

genes.granges <- GRanges(seqnames=df.genes.contig$CONTIG , ranges=genes.ranges, strand=as.factor(df.genes.contig$STRAND))

alns.granges <- GRanges(seqnames=df.alns.contig$REFERENCE_NAME, ranges=alns.ranges , strand=as.factor(df.alns.contig$STRAND))

# Number of sequences overlapping each gene

genes.contig.counts <- countOverlaps(query=genes.granges, subject=alns.granges,

type="any", minoverlap=1, ignore.strand=TRUE)

return(genes.contig.counts)

}))

## Gene abundances in the sample

df.genes[,SAMPLE] <- 0

df.genes[names(genes.counts),SAMPLE] <- genes.counts

## Normalize abundances by gene length

df.genes[,paste(SAMPLE,"NORM",sep="_")] <- round(df.genes[,SAMPLE]/df.genes$GENE_LENGTH, digits=4)

## Expressed as percentage

total <- sum(df.genes[,paste(SAMPLE,"NORM",sep="_")])

df.genes[,paste(SAMPLE,"NORM","PCT",sep="_")] <- round(df.genes[,paste(SAMPLE,"NORM",sep="_")]*100/total, digits=4)

}

## Remove "empty" rows

i <- which(apply(df.genes[,SAMPLES], MARGIN=1, function(x) {all(x==0)}))

df.genes <- df.genes[-i,]

## Write the genes abundance table

write.table(df.genes, file=file.path(WORKDIR,"genes.counts.tsv"),

quote=FALSE, sep="\t", row.names=FALSE, col.names=TRUE)
